# A basigin antibody modulates MCTs to impact tumor metabolism and immunity

**DOI:** 10.1038/s41421-025-00777-1

**Published:** 2025-05-06

**Authors:** Heng Zhang, Xuemei Yang, Yue Xue, Yi Huang, Yingxi Mo, Yurun Huang, Hong Zhang, Xiaofei Zhang, Weixin Zhao, Bin Jia, Ningning Li, Ning Gao, Yue Yang, Dongxi Xiang, Shan Wang, Yi Qin Gao, Jun Liao

**Affiliations:** 1 School of Life Science and Technology, ShanghaiTech University, Shanghai, China; 2 Beijing National Laboratory for Molecular Sciences, College of Chemistry and Molecular Engineering, Peking University, Beijing, China; 3 Department of Experimental Research, Guangxi Medical University Cancer Hospital, Nanning, Guangxi China; 4 Department of Radiation Oncology, Fudan University Shanghai Cancer Center, and Department of Oncology, Shanghai Medical College, Fudan University, Shanghai, China; 5 Lung Cancer Department, Tianjin Cancer Hospital, Tianjin, China; 6 State Key Laboratory of Membrane Biology, Peking-Tsinghua Joint Center for Life Sciences, School of Life Sciences, Peking University, Beijing, China; 7 Institute of Toxicology, School of Public Health, Lanzhou University, Lanzhou, Gansu China; 8 State Key Laboratory of Systems Medicine for Cancer, Shanghai Cancer Institute, and Department of Biliary-Pancreatic Surgery, the Renji Hospital Affiliated to Shanghai Jiaotong University School of Medicine, Shanghai, China; 9 Alphelix Biosciences, Foshan, Guangdong, China

**Keywords:** Targeted therapies, Cryoelectron microscopy

## Abstract

Lactate metabolism and signaling intricately intertwine in the context of cancer and immunity. Basigin, working alongside monocarboxylate transporters MCT1 and MCT4, orchestrates the movement of lactate across cell membranes. Despite their potential in treating formidable tumors, the mechanisms by which basigin antibodies affect basigin and MCTs remain unclear. Our research demonstrated that basigin positively modulates MCT activity. We subsequently developed a basigin antibody that converts basigin into a negative modulator, thereby suppressing lactate transport and enhancing anti-tumor immunity. Additionally, the antibody alters metabolic profiles in NSCLC-PDOs and T cells. Cryo-EM structural analysis and molecular dynamics simulations reveal that the extracellular Ig2 domain and transmembrane domain of basigin regulate MCT1 activity through an allosteric mechanism. The antibody decreases MCT1 transition rate by reducing the flexibility of basigin’s Ig2 domain and diminishing interactions between basigin’s transmembrane domain and MCT1. These findings underscore the promise of basigin antibodies in combating tumors by modulating metabolism and immunity, and the value of a common therapeutic subunit shared by multiple transporter targets.

## Introduction

Lactate and lactic acid, byproducts of the Warburg effect (aerobic glycolysis), play intricate roles in tumor development and the immune response^1–4^. Lactic acid promotes tumor progression by reprogramming energy metabolism, inducing angiogenesis, and suppressing immunity^1–4^. For example, elevated lactic acid levels provide regulatory T cells (Tregs) a metabolic edge, enabling them to maintain their suppressive function and promote tumor proliferation^5–7^. The proton-coupled monocarboxylate transporters (MCTs), specifically MCT1 and MCT4 (Supplementary Fig. S1), are crucial and complementary in managing cellular lactate dynamics^8^, with MCT1 handling both influx and efflux, and MCT4 primarily managing efflux. Clinical trials exploring specific MCT1 inhibitors to kill cancers by disrupting the cancer cells’ metabolism often faced primary resistance due to MCT4 upregulation^9^. Dual MCT1/4 inhibitors have been pursued but have not yet achieved success^10–12^.

Basigin/CD147 (BSG, Supplementary Fig. S2) is a single-pass transmembrane glycoprotein with a multifaceted role in cancer progression and metastasis^13^. BSG interacts with various transmembrane and soluble partners, including MCT1, MCT4, GLUT1, CD44, integrins, cyclophilins, matrix metalloproteinases (MMPs), caveolin-1, and CD276^13,14^. BSG serves as a chaperone for MCT1 and MCT4, ensuring their proper localization and function on the cell membrane while preventing their internalization and degradation^15–17^. When BSG expression is depleted or its interaction with MCT1/MCT4 is disrupted, the surface expression and stabilization of MCT1 and MCT4 are reduced, impairing the lactate shuttling crucial for tumor survival^11,18,19^. Diverse anti-BSG antibodies are being developed to combat cancers^18,20–27^, with some showing significant promise in challenging cancers like pancreatic ductal adenocarcinoma (PDAC) and hepatocellular carcinoma (HCC)^24–27^. However, it remains unclear how BSG acts on its partners, and the mechanisms by which BSG antibodies affect these partners are not well understood. This lack of clarity could hinder the further optimization and enhancement of therapies targeting BSG and its associated partners.

Here, we initially utilized a fluorescence-based flux assay and discovered that mouse BSG (mBSG) serves as a positive modulator, enhancing the activities of both mouse MCT1 (mMCT1) and mouse MCT4 (mMCT4). Subsequently, we identified a monoclonal antibody, 6E7F1 (Supplementary Fig. S3), which transforms mBSG into a negative modulator of mMCT1 and mMCT4. The 6E7F1 antibody impedes mMCT-mediated lactate transport, reduces cancer cell viability in vitro, and slows tumor growth in immunocompromised mice. Furthermore, the antibody activates immune responses in both immunocompetent mice and organoids (NSCLC-PDOs) derived from non-small cell lung cancer (NSCLC) patients, indicating its potential to reduce lactate shuttling essential for tumor survival and enhance immune responses in combating tumors.

Cryo-electron microscopy (cryo-EM) structures of mMCT1 in complex with mBSG and 6E7F1Fab at 5.0 Å resolution, along with the extracellular domain of mBSG in complex with 6E7F1Fab at 3.2 Å resolution, were examined. Through a combination of molecular dynamics (MD) simulations and biochemical assays, we unveiled that the extracellular Ig2 domain and transmembrane domain (TM) of mBSG orchestrate mMCT1 activity via an allosteric mechanism. 6E7F1, negatively modulates mMCT1 transition by decreasing the flexibility of mBSG’s Ig2 domain and weakening the interactions between mBSG’s TM and mMCT1. Our research highlights the benefits of using a shared subunit, like BSG, across key transporters such as MCT1 and MCT4, to suppress tumor survival and modulate immune responses.

## Results and discussion

### BSG functions as a positive MCT modulator

**Fig. 1:**
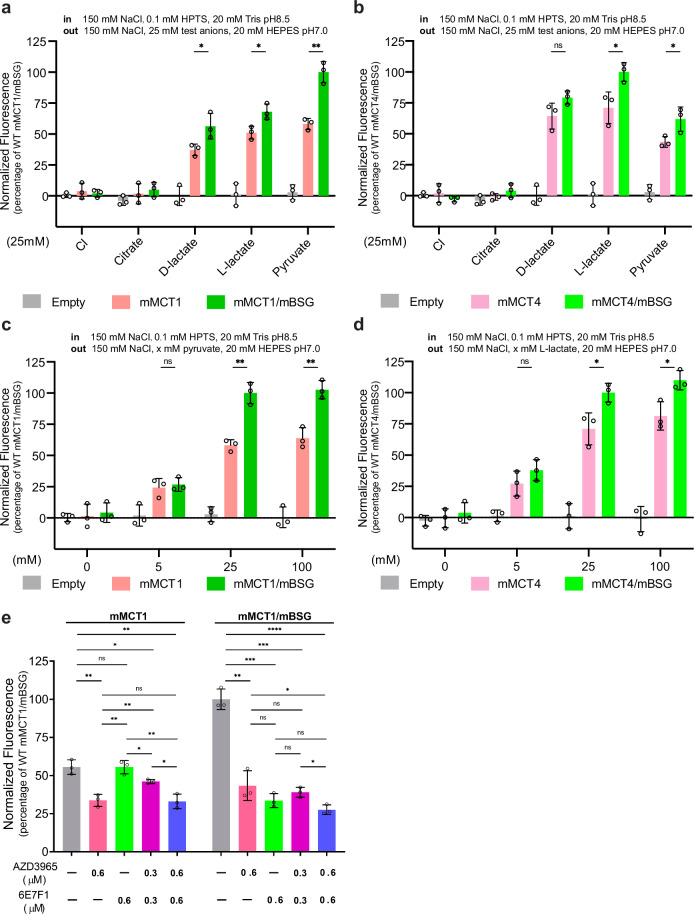
mBSG accelerates MCT-mediated H^+^/monocarboxylate symport. **a** Transport activities of mMCT1 and mMCT1/mBSG in the presence of test anions. Transport activities are determined using the liposomal flux assay, in which normalized fluorescence quenching of the pH-sensitive dye, HPTS, is measured. Data are scaled to the normalized fluorescence quenching of WT mMCT1/mBSG in the presence of 25 mM extraliposomal pyruvate, pH 7.0 (**a**, **c**), or the normalized fluorescence quenching of WT mMCT4/mBSG in the presence of 25 mM extraliposomal L-lactate, pH 7.0 (**b**, **d**). “Empty” refers to control protein-free liposomes. All test anions are sodium salts. **b** Transport activities of mMCT4 and mMCT4/mBSG in the presence of test anions. **c** mMCT1- or mMCT1/mBSG-mediated H^+^/pyruvate influx in response to different extraliposomal pyruvate concentrations. **d** mMCT4- or mMCT4/mBSG-mediated H^+^/L-lactate influx in response to different extraliposomal L-lactate concentrations. **e** mMCT1- or mMCT1/mBSG-mediated H^+^/pyruvate influx in response to 6E7F1, and/or AZD3965. Intraliposomal buffer: 150 mM NaCl, 0.1 mM HPTS, 20 mM Tris, pH 8.5; extraliposomal buffer: 150 mM NaCl, 25 mM sodium pyruvate, 20 mM HEPES, pH 7.0. IgG and/or DMSO are controls for 6E7F1 and/or AZD3965, respectively.

We investigated the transport activities of mMCT1 and mMCT4 using a fluorescence-based assay^28^ (Fig. 1a–d; Supplementary Figs. S4 and S5a). Briefly, liposomes containing mMCT1 or mMCT4 were loaded with a pH-sensitive dye 8-Hydroxypyrene-1,3,6-trisulfonic acid (HPTS) at pH 8.5. By adding a test anion to an extraliposomal buffer at pH 7.0, we initiated H^+^ influx. If the test anion is a substrate for mMCT1 or mMCT4, the H^+^ gradient across the liposomal lipid bilayer drives both H^+^ and the test anion into the liposome, resulting in a decrease in intraliposomal pH. This pH change is monitored by H^+^-induced fluorescence quenching of HPTS. Protein-free (termed empty) liposomes were analyzed in parallel as a control (Fig. 1a–d; Supplementary Figs. S4 and S5a). As expected, empty liposomes showed negligible fluorescence quenching, indicating minimal leakage of H^+^ and test anions across the lipid bilayer. For mMCT1-containing liposomes, chloride or citrate did not cause notable fluorescence quenching (Fig. 1a, b; Supplementary Figs. S4a and S5a), suggesting that these anions do not pass through these transporters. By contrast, short-chain monocarboxylates (like D- and L-lactates, and pyruvate) induced H^+^ influx across mMCT-containing liposomes (Fig. 1a, b; Supplementary Figs. S4a and S5a), confirming their permeability. Notably, mMCT1 and mMCT4 were most selective for pyruvate and L-lactate, respectively, consistent with previous findings^8^. Higher pyruvate and L-lactate gradients drove more H^+^ influx (Fig. 1c, d) and vice versa (Supplementary Fig. S4b), indicating that either monocarboxylate or H^+^ gradients can power these transporters^8^. We then performed our flux assay using mMCT1/mBSG- or mMCT4/mBSG-containing proteoliposomes, which clearly demonstrated that mBSG increased the transporter activities of both mMCT1 and mMCT4 (Fig. 1a–d; Supplementary Figs. S4a, b and S5a). Together, our fluorescence-based assays confirmed that mMCT1 and mMCT4 are selective symporters for monocarboxylates like pyruvate and L-lactate. Additionally, mBSG not only acts as their chaperone but also enhances their activities. This inspired the creation of an mBSG-targeting mMCT modulator antibody 6E7F1.

### A novel antibody 6E7F1 converts BSG into a negative MCT modulator

We investigated whether mBSG could be transformed into a negative modulator using antibodies. Using a protocol that combines the enzyme-linked immunosorbent assay (ELISA) and the aforementioned fluorescence-based flux assay, we found a monoclonal antibody, 6E7F1 (Supplementary Fig. S3), which specifically binds to the Ig2 domain of mBSG (Supplementary Fig. S5b). When bound by 6E7F1, mBSG acted as a negative modulator for mMCT1 (Fig. 1e) and mMCT4 (Supplementary Fig. S5c), significantly reducing their transport activity by ~75% and ~50%, respectively. We investigated whether 6E7F1 inhibits MCTs as effectively as other known MCT blockers. We compared it to AZD3965, a potent MCT1 inhibitor that has been investigated in clinical trials^29^. 6E7F1 and AZD3965 showed similar inhibitory effects on mMCT1/mBSG-mediated pyruvate transport at equivalent concentrations (Fig. 1e). Additionally, combining half concentrations of allosteric 6E7F1 and orthosteric AZD3965 achieved similar efficacy as full concentrations of individual inhibitors (Fig. 1e), offering an alternative MCT1 inhibition strategy.

### BSG antibody 6E7F1 curbs tumor growth and enhances T cell immunity in vivo

**Fig. 2:**
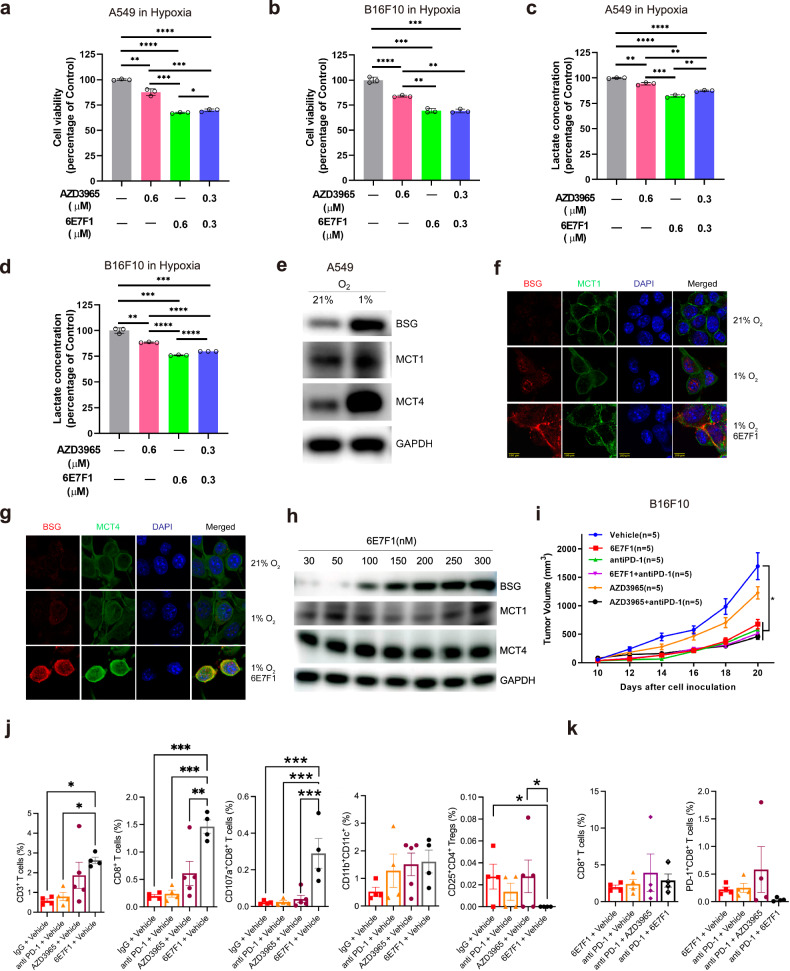
6E7F1 converts mBSG into a negative MCT modulator, suppressing tumor growth and enhancing T cell immunity. **a**, **b** Viability of human A549 lung carcinoma (**a**) and murine B16F10 melanoma (**b**) cells under hypoxia (1% O_2_) treated with 6E7F1 and/or AZD3965. **c**, **d** Extracellular lactate concentrations in cultured A549 (**c**) and B16F10 (**d**) cells under hypoxia, treated with 6E7F1 and/or AZD3965. **e** Western blot analysis of BSG, MCT1, and MCT4 protein levels in A549 cells under both normoxic and hypoxic conditions. **f**, **g** Immunofluorescent imaging reveals co-localization of BSG with MCT1 (**f**) and MCT4 (**g**) on the plasma membrane of A549 cells. **h** Western blot analysis of BSG, MCT1, and MCT4 protein levels in A549 cells under hypoxia with increasing concentrations of 6E7F1. **i** The effect of 6E7F1, AZD3965, and/or PD-1 antibody on B16F10 allograft tumor growth in immunocompetent C57BL/6 mice. **j**, **k** FACS analysis of tumor-infiltrating lymphocytes following specified administrations in immunocompetent C57BL/6 mice bearing B16F10 tumors.

We further tested the suppressive effects of 6E7F1 and AZD3965 on lactate transport and cancer cell survival in vitro. We selected human A549 NSCLC cells, murine B16F10 melanoma cells, and murine A20 lymphoma cells. The first two types are highly glycolytic under hypoxia^30,31^, while the third remains glycolytic even under normoxia^32^. Under hypoxia, treatment with 6E7F1 and/or AZD3965 reduced cell viability (Fig. 2a, b; Supplementary Fig. S6a) and extracellular lactate concentration (Fig. 2c, d) for both A549 and B16F10 cells. However, this effect was not observed under normoxia (Supplementary Fig. S6a). In contrast, suppressive effects of 6E7F1 and/or AZD3965 were seen in glycolytic A20 cells under normoxia (Supplementary Fig. S6b–d). Notably, 6E7F1, which was originally raised against mBSG, inhibited both human (A549) and murine (B16F10 and A20) cancer cells. This could be attributed to its cross-binding to human BSG (hBSG, Supplementary Fig. S7a, b).

A549 and B16F10 cells express both MCT1 and MCT4^30,31^. We confirmed this for A549 cells (Fig. 2e–g). Similarly, A20 cells also express MCT1 and MCT4 (Supplementary Fig. S6e). As expected, 6E7F1 exhibited more potent inhibitory effects than AZD3965 in suppressing lactate transport and cancer cell survival (Fig. 2a–d; Supplementary Fig. S6c, d), as 6E7F1 acts as a dual MCT inhibitor. Interestingly, our immunofluorescent staining and western blot analysis showed that the anticancer activity of 6E7F1 does not involve the withdrawal of MCT1 and MCT4 from the plasma membrane (Fig. 2f–h; Supplementary Fig. S6e). This mechanism is distinct from those that either reduce MCT1 and MCT4 surface expression/localization by silencing BSG expression or prevent BSG–MCT1 complex maturation by disrupting the interaction between cereblon (CRBN) and the BSG–MCT1 complex^11,19^.

We knocked down the expression of MCT1 and BSG using siRNAs and investigated how the reduced expression of these proteins affects the anticancer effects of AZD3965 and 6E7F1 under hypoxic conditions (Supplementary Fig. S6f–h). Our findings revealed that when MCT1 levels decreased due to siMCT1 treatment (Supplementary Fig. S6f), cancer cells became more resistant to AZD3965 (Supplementary Fig. S6g). Similarly, reduced BSG levels by siBSG treatment conferred resistance to 6E7F1 treatment (Supplementary Fig. S6f, h). These results demonstrated that the effectiveness of AZD3965 and 6E7F1 in killing cancer cells is linked to the expression levels of MCT1 and BSG proteins, respectively. Furthermore, reducing BSG in A549 cells using siRNA coincided with a reduction in MCT1 (Supplementary Fig. S6f), similar to the observations in other mammalian cells^11,16^. Consequently, AZD3965 exhibited similar effects on cells treated with either siBSG or siMCT1 (Supplementary Fig. S6g). In contrast, the dual MCT inhibitor, 6E7F1, demonstrated greater potency than AZD3965 when cells were treated with either siMCT1 or siMCT4 (Supplementary Fig. S6g, h). Interestingly, both AZD3965 and 6E7F1 exhibited the most potent cytotoxic effects on cells treated with siMCT4 (Supplementary Fig. S6g, h), underscoring the complementary roles of MCT1 and MCT4 in tumor survival^11^. We further evaluated the anti-tumor capability of 6E7F1 using an A549 xenograft tumor model in immunocompromised BALB/c nude mice (Supplementary Fig. S6i, j). Remarkably, 6E7F1 effectively reduced A549 tumor growth while having minimal impact on body weight.

Recent research highlights the pivotal role of tumor-associated lactic acid metabolism in orchestrating immunosuppression within the tumor microenvironment (TME)^6,7,33–36^. This metabolic process recruits tumor-associated macrophages, reduces cytotoxic CD8^+^ T cell amplification, and promotes intratumor Treg cell proliferation, ultimately creating an immunosuppressive environment that supports tumor growth. In a prior study using a B16F10 allograft tumor model in immunocompetent C57BL/6 mice, tumor-targeted inhibition of MCT1 by AZD3965 improved T-cell immunity against tumor by countering the immunosuppressive effects of the TME^29^. To gain further insights into how inhibiting both MCT1 and MCT4 impacts T-cell immunity, we examined the anti-tumor efficacy of AZD3965, 6E7F1, and PD-1 antibody, both individually and in combination, in B16F10 tumor-bearing C57BL/6 mice (Fig. 2i–k; Supplementary Fig. S6k), following a protocol similar to that described in the prior study^29^. Notably, 6E7F1 exhibited greater potency than AZD3965 in curbing tumor growth (Fig. 2i) and no significant toxicity in mice (Supplementary Fig. S6l). Flow cytometry (FACS) analysis revealed significant alterations in the TME’s cellular composition with 6E7F1 treatment compared to AZD3965 treatment (Fig. 2j, k). Specifically, 6E7F1 increased the proportions of CD3^+^ T cells, CD8^+^ T cells, CD8^+^CD107a^+^ cytotoxic T cells, and CD11c^+^CD11b^–^ dendritic cells (DCs), while reducing the proportions of CD4^+^CD25^+^ Treg cells. These findings highlighted the immunomodulatory impact of targeting BSG in the context of tumor immunity. Interestingly, inhibiting BSG appears to enhance effector T cell activity (specifically, CD8^+^CD107a^+^ T cells), while dampening suppressive functions mediated by CD8^+^PD-1^+^ T cells and Treg cells. The combinatory antibody treatment against BSG and PD-1 molecules appeared to decrease the proportion of CD8^+^PD-1^+^ cells, although no statistically significant difference was observed compared to single-treated counterparts or combinational controls (Fig. 2k). Together, our findings underscore the efficacy of the dual MCT1 and MCT4 inhibitor 6E7F1 in inhibiting lactate transport and tumor growth.

### BSG antibody 6E7F1 suppresses NSCLC-PDO growth

**Fig. 3:**
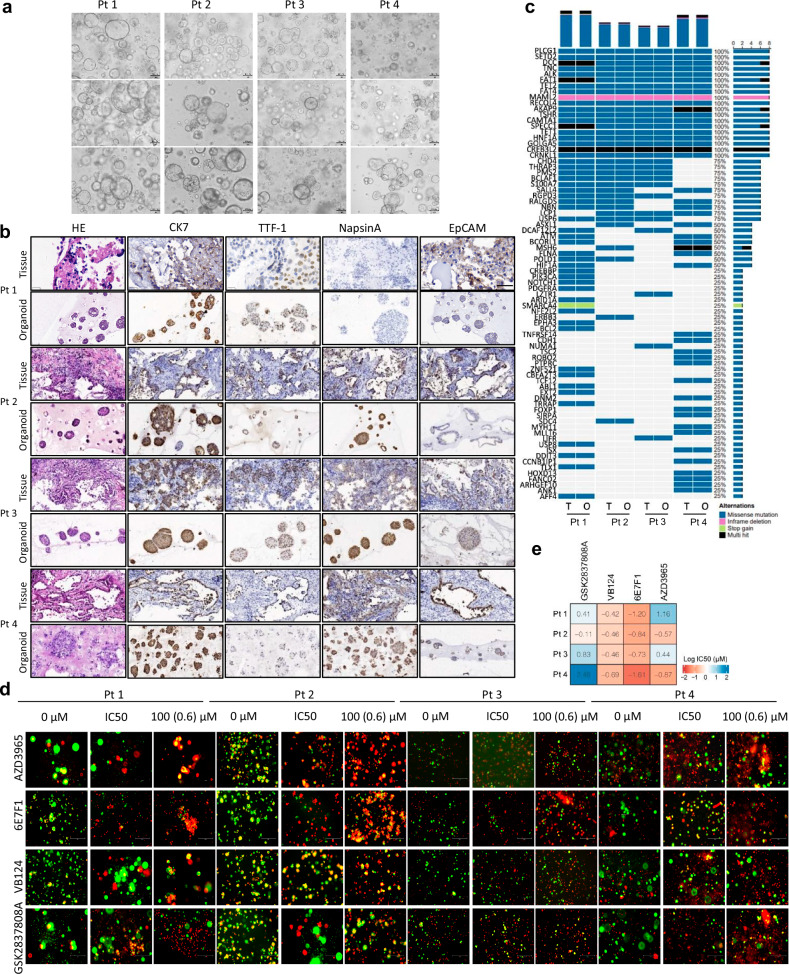
BSG antibody 6E7F1 suppresses the growth of NSCLC-PDOs. **a** Images of NSCLC-PDOs derived from four NSCLC patients (Pt 1, Pt 2, Pt 3, and Pt 4). Scale bars, 100 µm. **b** HE and IHC staining of NSCLC biopsies and their corresponding organoids. Pathological markers for IHC staining included CK7, TTF-1, NapsinA, and EpCAM. Scale bars, 50 µm. **c** Genetic variants identified in each sample based on WES analysis. The color reflects correlation. T: tumor tissue; O: organoid. **d** Sensitivity of the four NSCLC-PDOs to glycolysis inhibitors. The highest concentrations were up to 100 µM for small molecules and 0.6 µM for 6E7F1. Dead cells and living cells were stained by propidium iodide (PI, red) and Calcein-AM (green), respectively. **e** Heatmap depicting the responses of NSCLC-PDOs to drugs, including GSK2837808A, VB124, 6E7F1, and AZD3965. The color scale represents average LogIC_50_ values.

We further employed NSCLC-PDOs (Patient 1(Pt 1), Pt 2, Pt 3, and Pt 4) (Fig. 3a; Supplementary Fig. S8) to assess the potential anti-tumor effects of 6E7F1 in clinical contexts. We conducted hematoxylin and eosin (HE) and immunohistochemistry (IHC) staining to evaluate the biological fidelity of organoids to their tissue origins, which showed consistent pathological morphology and protein expression (Fig. 3b). Whole-exosome sequencing (WES) analysis further confirmed the retention of key gene mutations in organoids (Fig. 3c), suggesting genetic alignment between organoids and NSCLC tissues. To evaluate the impact of targeting lactic acid metabolism on organoid growth, we administered 6E7F1, AZD3965, VB124 (an MCT4 inhibitor), and GSK2837808A (a lactate dehydrogenase A (LDHA) inhibitor) to four NSCLC-PDOs (Fig. 3d, e; Supplementary Fig. S9). Notably, all NSCLC-PDOs exhibited greater sensitivity to 6E7F1 than to the other three drugs, indicating that simultaneous inhibition of cross-membrane lactic acid transport via MCT1 and MCT4 outperformed individual inhibition of MCT1 or MCT4, as well as LDHA-mediated lactic acid production.

### BSG antibody 6E7F1 modulates the metabolism of NSCLC-PDOs and T cells

**Fig. 4:**
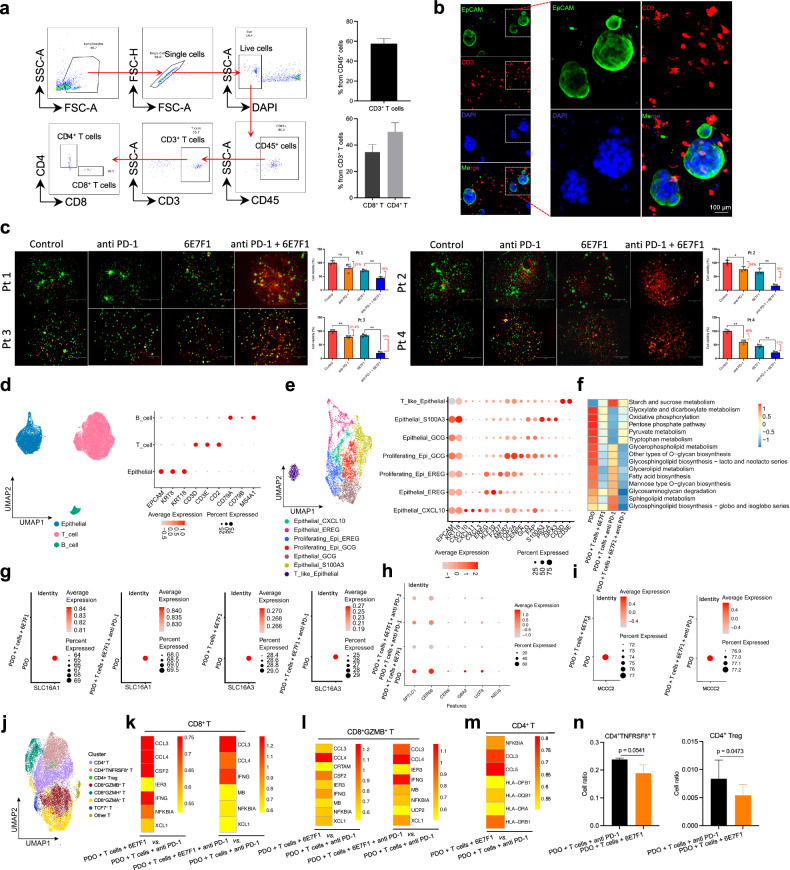
BSG antibody 6E7F1 modulates the metabolism of the NSCLC-PDOs and T cells. **a** Representative FACS analysis of co-cultured tumor organoids and T cells on Day 12 (left panel) and the statistics of T cell composition (right panel). **b** Representative images of co-cultured tumor organoids and T cells. **c** Representative images of co-cultured NSCLC-PDOs and T cells (left panel) and the statistics of each PDO’s viability (right panel) under treatments with control phosphate-buffered saline (PBS), PD-1 antibody, 6E7F1, and a combination of the PD-1 antibody and 6E7F1. **d**, **e** UMAP plots illustrating cell types and classical marker genes for the co-cultured NSCLC-PDOs and T cells (**d**), as well as epithelial cells (**e**). **f** Heatmaps illustrating metabolic variation within epithelial groups under the specified treatments. **g** Dot plots displaying the transcriptional variation of *SLC16A1* and *SLC16A3* within epithelial groups under the specified treatments. **h**, **i** Dot plots showing the expressional variation of genes involved in the sphingolipid pathway (**h**) or the *MCCC2* gene (**i**) within the epithelial groups under the specified treatments. **j** UMAP plot depicting various categories of T cells. **k**, **l** Heatmaps depicting the expression variability of genes in CD8^+^ T cells (**k**) and in CD8^+^GZMB^+^ T cells (**l**) under specified treatments. The Seurat indicates average gene expression. **m** Heatmap depicting the expression variability of genes in CD4^+^ T cells under specified treatments. **n** The population variation of CD4^+^TNFRSF8^+^ T cells (left panel) and CD4^+^ Treg cells (right panel) under specified treatments.

We co-cultured NSCLC-PDOs with T cells to explore how inhibiting lactic acid flux and blocking PD-1 impact tumor immunity, with a specific focus on metabolic profiles. To validate the co-culture system, we examined the presence and composition of T cells on Day 12 following the co-culture of tumor organoids with immune cells. Approximately 60% of the CD45^+^ cells were T cells, with the majority identified as CD3^+^ T cells, comprising both CD8^+^ and CD4^+^ subsets (Fig. 4a). Furthermore, immunostaining demonstrated direct interactions between tumor organoids and T cells (Fig. 4b). Using 6E7F1 and PD-1 antibodies, alone or combined, we observed significant organoid cell death (Fig. 4c). As expected, the combined treatment outperformed individual treatments. Subsequently, we used single-cell RNA sequencing (scRNA-seq) to identify three cell types in the co-cultures: epithelial cells (*EPCAM*, *KRT8*, and *KRT18*), T cells (*CD3D*, *CD3E*, and *CD2*), and a minor population of B cells (*CD79A*, *CD79B*, and *MS4A1*) (Fig. 4d). Epithelial cells exhibited seven distinct subtypes (Fig. 4e), showing increased proliferation and elevated expression of *EREG*, *CXCL10*, and *GCG*, suggesting stem-like properties in the organoid system. When treated with either 6E7F1 or a combination of 6E7F1 and PD-1 antibodies, the transcription levels of *SLC16A1* (*MCT1*) and *SLC16A3* (*MCT4*) were reduced (Fig. 4g). Whether MCT1 and MCT4 protein expression is also reduced in NSCLC-PDOs warrants further investigation.

NSCLC-PDOs treated with 6E7F1 displayed pronounced alterations in metabolic pathways including starch and sucrose metabolism, glycerophospholipid metabolism, glycerolipid metabolism, fatty acid biosynthesis, glycosaminoglycan degradation, and sphingolipid metabolism (Fig. 4f; Supplementary Fig. S10). Notably, key genes involved in sphingolipid metabolism, such as *SPTLC1*, *CERS6*, *CERK*, *GBA2*, *UGT8*, and *NEU3*, were downregulated (Fig. 4h). Components of the sphingolipid pathway, including sphingosine-1-phosphate (S1P) and ceramide, reciprocally regulate cell fate^37,38^. Ongoing clinical anti-cancer trials explore strategies that inhibit pro-survival S1P signaling while enhancing anti-proliferative ceramide signaling^37–40^. Moreover, elevated expression of methylcrotonoyl-CoA carboxylase 2 (MCCC2), a key enzyme involved in the breakdown of leucine and isovaleric acid, has been linked to oncogenic processes in HCC^41^, colorectal cancer (CRC)^42^, and breast cancer^43^. Intriguingly, 6E7F1 treatment led to a significant reduction in *MCCC2* expression (Fig. 4i, left panel), which is further potentiated when combined with PD-1 antibodies (Fig. 4i, right panel). These findings suggest that 6E7F1 exerts its anti-tumor effect by influencing leucine metabolism and fatty acid/lipid pathways.

Our study has systematically classified T cells into eight distinct subtypes (Fig. 4j). When comparing PD-1 antibody treatment to treatments involving 6E7F1 (alone or in combination with PD-1 antibody), we observed upregulated expression of *IFNG*, *CCL4*, and *XCL1* genes in CD8^+^ T cells (Fig. 4k). This effect was particularly notable in the CD8^+^GZMB^+^ T cell subset (Fig. 4l). The secretion of XCL1 by antigen-specific CD8^+^ T cells, along with IFNγ, suggests that 6E7F1 treatment may enhance cross-presentation and antigen uptake by T cells^44^, ultimately promoting adaptive cytotoxic immunity. Low expression of HLA class II antigens (such as HLA-DPB1, HLA-DRA, and HLA-DQB1) has been linked to tumor malignancy^45^. Conversely, upregulated CD4^+^ T cell-related HLA class II expression contributes to improved clinical efficacy in PD-L1/PD-1 blockade therapies, enhancing anti-tumor immunity^46^. In our study, we observed HLA class II upregulation in CD4^+^ T cells following 6E7F1 treatment (Fig. 4m). Additionally, TNFRSF8, in conjunction with FOXP3 and CTLA4, serves as a marker for identifying tumor-infiltrating Tregs that play a role in tumor localization and evasion^47–49^. 6E7F1 treatment reduced the population of both CD4^+^TNFRSF8^+^ T cells and Tregs (Fig. 4n). These findings suggest that BSG antibody therapy holds promise for boosting anti-tumor immune responses.

### The overall cryo-EM structure of mMCT1/mBSG/6E7F1Fab complex

**Fig. 5:**
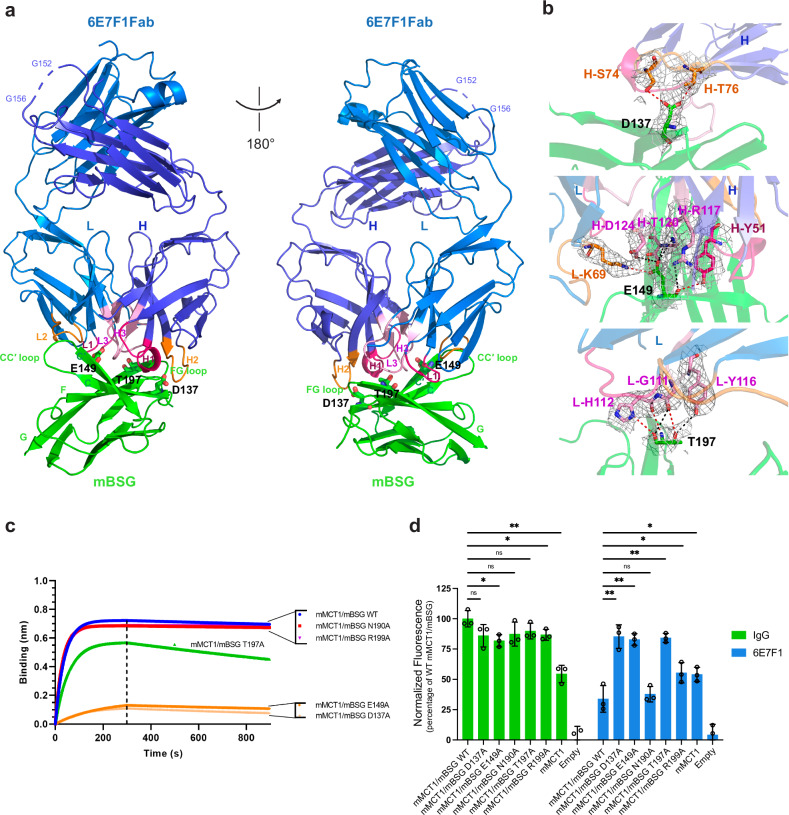
6E7F1’s interactions with mBSG impact its ability to inhibit mMCT1/mBSG activity. **a** Overview of the interaction between 6E7F1Fab and the Ig2 domain of mBSG. Three mBSG residues (D137, E149, and T197), each of which interact with multiple 6E7F1Fab residues, are marked. **b** Zoomed-in view of interactions between 6E7F1Fab residues and D137 (top), E149 (middle), and T197 (bottom) of mBSG. Cryo-EM density (grey, 5.0 σ) of interacting residues is shown. Red dashed lines represent H-bonds (bond length of 2.5–3.5 Å); black dashed lines indicate distances between two polar atoms (O or N) (3.6–4.5 Å). **c** The binding affinity of 6E7F1 to WT mMCT1/mBSG or mMCT1/mBSG variants measured by Octet biolayer interferometry. Purified 6E7F1 was immobilized on Anti-Mouse IgG Fc Capture biosensors. Each analyte is 200 nM. **d** The transport activity of mMCT1 affected by the binding affinity of 6E7F1 to WT mMCT1/mBSG and variants. Intraliposomal buffer: 150 mM NaCl, 0.1 mM HPTS, 20 mM Tris, pH 8.5; extraliposomal buffer: 150 mM NaCl, 25 mM pyruvate, 20 mM HEPES, pH 7.0.

To comprehend how BSG and its antibody impact MCT activity, we embarked on resolving the cryo-EM structure of the antigen binding fragment of 6E7F1 (referred to as 6E7F1Fab), in conjunction with mMCT1/mBSG. Unfortunately, due to the dynamic interplay between the TM segment and the extracellular soluble segment of mBSG, we could not unveil the entire complex. Instead, we independently resolved two distinct parts from separate data sets (Supplementary Fig. S11a–i, and Table S1): the TM part (including mBSG’s single TM (termed TM0) and twelve TMs of mMCT1) at 5.0-Å resolution (Supplementary Fig. S11a–d, j), and the extracellular region of mBSG along with the bound 6E7F1Fab at 3.2-Å resolution (Fig. 5a, b; Supplementary Figs. S11e–i, S12 and S13). The latter allowed us to construct an accurate atomic model, encompassing mBSG’s Ig2 domain (I99–R209) and the heavy (Q20–R236) and light (D21–E233) chains of 6E7F1Fab (Supplementary Figs. S2 and S3). We then pieced together the overall structure of the mMCT1/mBSG/6E7F1Fab complex from these two resolved segments (Supplementary Fig. S11k–m).

The atomic model of mMCT1/mBSG/6E7F1Fab complex (Supplementary Fig. S11k) exhibits a distinctive ‘tobacco pipe’ shape, with mMCT1 resembling a bowl and mBSG/6E7F1Fab resembling a shank. mMCT1 comprises N-terminal (NTD, TM1–TM6) and C-terminal (CTD, TM7–TM12) domains (Supplementary Fig. S1), adopting an outward-open conformation akin to hMCT1 bound to lactate (PDB: 6LZ0)^50^. The Ig2 domain of mBSG (Fig. 5a; Supplementary Fig. S12a–d), part of the I-set immunoglobulin superfamily (IgSF), consists of two β-sheets (DEBA and A′GFCC′) connected by a conserved disulfide bond^51^. Notably, strands A and A′ within the I99–G119 segment exhibit intriguing structural features (Supplementary Fig. S12c). They engage in extensive hydrogen-bond (H-bond) interactions between strands A and B (left panel) and between strands A′ and G (right panel), as well as hydrophobic interactions between strands A and G (right panel). These interactions suggest that strands A and A′ act as a connecting joint between the β-sheets DEBA and A′GFCC′, influencing the Ig2 domain’s flexibility.

### 6E7F1’s interactions with mBSG impact its ability to inhibit mMCT1/mBSG activity

6E7F1Fab adopts a typical immunoglobulin fold (Fig. 5a; Supplementary Fig. S12e). Its VH and VL domains are situated on a concave surface formed by the CC′ and FG loops, along with strands C, C′, F, and G within the Ig2 domain of mBSG (Fig. 5a). The conservation of interfacial residues among mBSG orthologs (Supplementary Fig. S2) and the structural similarity (Supplementary Fig. S12b) between the Ig2 domains of mBSG and hBSG^51^ (PDB: 3B5H) may explain why 6E7F1 can bind to both BSGs (Supplementary Fig. S7a, b).

To assess whether 6E7F1’s binding affinity to mBSG affects its ability to inhibit mMCT1/mBSG activity, we introduced alanine mutations in mBSG at key residues involved in antibody/mBSG interactions (Fig. 5b; Supplementary Figs. S2 and S3). These residues included: 1) D137 in strand C, which forms H-bonds with S74 and T76 in the H2 of 6E7F1Fab, 2) E149 in strand C′, which forms H-bonds with Y51 in H1, with R117, T120, and D124 in H3, and with K69 in L2 of 6E7F1Fab, and 3) T197 in strand G, which forms H-bonds with G111, H112, and Y116 in L3 of 6E7F1Fab. The affinities of 6E7F1 for these alanine variants (mMCT1/mBSG D137A, E149A, T197A) were significantly reduced compared to wild-type (WT) mMCT1/mBSG (Fig. 5c; Supplementary Fig. S7c, d). While 6E7F1 only slightly inhibited the mMCT1 transport activity of these variants, WT mMCT1/mBSG and other high-affinity variants (e.g., mMCT1/mBSG N190A and mMCT1/mBSG R199A) showed significantly reduced mMCT1 transport activities in its presence (Fig. 5c, d; Supplementary Fig. S7c, d). These findings highlight the importance of specific residues at the 6E7F1–mBSG interface for tight binding and effective inhibition of mMCT1 transport activity.

### The mechanism by which mBSG accelerates mMCT1 transition

**Fig. 6:**
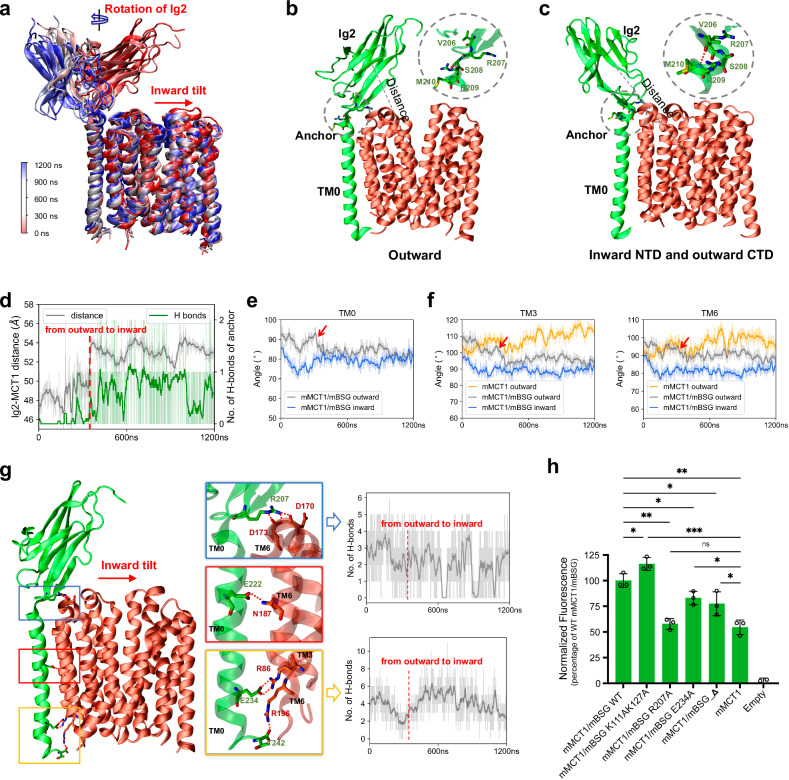
The mechanism by which mBSG accelerates mMCT1 transition. **a** Spontaneous evolution of the mMCT1/mBSG structure in a 1200 ns trajectory initiated with the outward-open mMCT1/mBSG. Analyses in **b**–**g** are based on the same trajectory. **b**, **c** Representative snapshots from the MD trajectory of mMCT1/mBSG, showing two conformations: an outward-facing conformation (**b**) and a conformation with an inward-facing NTD and outward-facing CTD (**c**). The center-to-center distance between the Ig2 domain and mMCT1 is indicated by a grey dashed line. Zoomed-in views of the anchor (V206–M210) of mBSG are shown in the upper right insets for each model. **d** Time-evolved changes in the center-to-center distance between the Ig2 domain and mMCT1 (grey), and the number of H-bonds (green) within the anchor of mBSG over the trajectory. In **d**–**f**, individual data points are plotted as light, transparent traces, while average values are represented by bold traces. **e**, **f** Time-evolved changes in the angles of TM0 within mBSG (**e**), and TM3 and TM6 in the NTD of mMCT1 (**f**) relative to the membrane surface over the trajectory. The red arrow indicates the transition from an outward- to an inward-facing conformation. **g** Time-evolved changes of three sets of H-bonds over the trajectory. Shown are a representative snapshot of the simulation model (left), zoomed-in views (middle) of these H-bonds, and time-evolved changes (right) to the extracellular and intracellular H-bonds. **h** Validation of the impact of the Ig2 domain and H-bond interactions between mBSG’s TM0 and mMCT1’s NTD on mMCT1 transport activity. The mMCT1/mBSG_∆ variant has truncated mBSG with residues M1–L204.

To understand how mBSG potentiates mMCT1 transport activity, we performed extensive MD simulations. These simulations, starting from the outward-open mMCT1/mBSG conformation and spanning 1200 ns each, revealed significant mBSG Ig2 domain mobility (Fig. 6a; Supplementary Video S1). That is, the Ig2 domain’s proximity to mMCT1 can be dynamically adjusted by rotating around a perpendicular axis to the membrane plane (Fig. 6a–d). This rotation of Ig2 is associated with a remarkable conformational change in the linker segment V206–M210 (referred to as ‘anchor’; V206 and R207 are the C-terminal residues of the Ig2 domain) between the TM0 and the Ig2 domains. Specifically, it transitions from a β-strand to an α-helix configuration as the Ig2 domain moves from a mMCT1-proximal to a mMCT1-distal position (Fig. 6b–d). Additionally, the mBSG’s TM0 tilts ~10°, resembling the configuration seen in the inward-facing mMCT1/mBSG structure (Fig. 6c, e; Supplementary Fig. S14a, b). Interestingly, a similar inward tilt of the BSG TM0 was observed in the cryo-EM structure of the human phospholipid scramblase Xkr-8/BSG complex in an inward-facing state^52^. Collectively, these findings underscored the dynamic mobility of the mBSG Ig2 domain, which can rotate away from mMCT1, ultimately inducing an outward-to-inward tilt of TM0.

Our MD simulations revealed three persistent H-bond sets connecting mBSG’s TM0 to mMCT1’s NTD in the simulation trajectories (Fig. 6g; Supplementary Figs. S1, S2 and S14c): extracellular side (mBSG’s R207 and mMCT1’s D170 and D173), within the membrane (mBSG’s E222 and mMCT1’s N187), and intracellular side (mBSG’s E234 and mMCT1’s R86, and mBSG’s T242 and mMCT1’s R196). Notably, the H-bond within the membrane plays a critical role in stably anchoring MCT1 to the plasma membrane^15,16,29^. Owing to the tight bond between TM0 of mBSG and the NTD of mMCT1, an inward tilt of TM0 could prompt NTD transitioning from an outward- to an inward-facing conformation (Fig. 6f; NTD is represented by its TM3 and TM6 for simplicity in Fig. 6f; Supplementary Fig. S14a, b and Video S1). Meanwhile, the CTD of mMCT1 maintains its outward-facing conformation in the simulation trajectories (Fig. 6c; Supplementary Fig. S14a, b). Mechanistically, we speculated that the simulated mMCT1 structure — an NTD facing inward and a CTD facing outward — may serve as an intermediate state poised for the transition to an inward-facing state upon binding of substrates like H^+^ or monocarboxylates.

To further validate mBSG’s impact on mMCT1, we conducted three parallel MD simulations for mMCT1 alone, beginning from the outward-facing state (Fig. 6f; Supplementary Fig. S14a). As anticipated, in the absence of mBSG, mMCT1 consistently maintained its outward-facing conformation and rarely transitioned to an intermediate state conducive to the outward-to-inward transition.

We functionally tested our simulations by generating mBSG variants and assessing their influence on mMCT1 transport activity (Fig. 6h; Supplementary Fig. S15a, c–f). Firstly, we generated the mMCT1/mBSG_∆ variant by deleting residues 1–204 (Ig1 and Ig2 domains) while retaining the anchor up to mBSG’s CTD. As expected, this variant exhibited reduced transport activity compared to WT mMCT1/mBSG. Secondly, we created R207A and E234A variants that impair the H-bond interactions between mBSG’s TM0 and mMCT1’s NTD at the extracellular and intracellular sides, respectively. These mMCT1/mBSG variants exhibited reduced transport activity compared to WT mMCT1/mBSG. Thirdly, we introduced the K111A mutation to the I99–G119 segment and the K127A mutation to strand B of the mBSG’ Ig2 domain. MD simulations predicted that these mutations increased Ig2 domain flexibility due to a decreased proportion of β-strands and reduced interactions between I99–G119 and strand B (Supplementary Fig. S15a, c, d), and an enhanced extracellular H-bond interactions between mBSG’s TM0 and the NTD of mMCT1 (Supplementary Fig. S15e). Consequently, the mMCT1/mBSG (K111A/K127A) variant exhibited a higher activity than the WT complex (Fig. 6h). Together, our simulations and functional analyses highlight the crucial role of Ig2 domain mobility and the strong bonds between mBSG’s TM0 and mMCT1’s NTD in driving the mMCT1 transition within mMCT1/mBSG complex.

### The mechanism by which 6E7F1 inhibits mMCT1

**Fig. 7:**
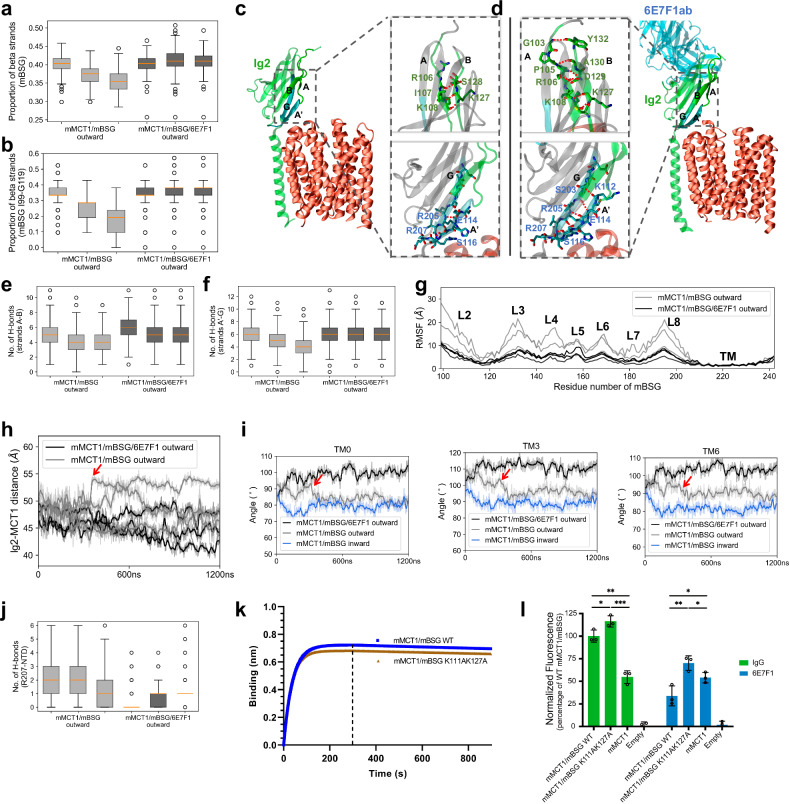
Mechanism by which 6E7F1 inhibits mMCT1. **a**, **b** Boxplots of the proportion of β-strands within mBSG (**a**) and its I99–G119 segment (**b**) over trajectories. For boxplots in **a**, **b**, **e**, **f** and **j**, the box extends from the first quartile (Q1) to the third quartile (Q3) of the data, with an orange line at the median. The whiskers extend from the box by 1.5× inter-quartile range (IQR). Outliers beyond the extreme ends of the whiskers are represented as individual points. **c**, **d** Snapshots from trajectories for mMCT1/mBSG (**c**) and mMCT1/mBSG/6E7F1Fab (**d**). H-bond interactions between strands A and B (top) and between strands A′ and G (bottom) are highlighted in zoomed-in views. **e**, **f** Boxplots of the number of H-bonds between strands A and B (**e**), and between strands A′ and G (**f**) over trajectories. **g** RMSF values for the Cα atoms of residues in mBSG across the trajectories. **h** Changes in the center-to-center distance between the Ig2 domain and mMCT1 over the trajectories. In **h** and **i**, individual data points are plotted as light, transparent traces, while average values are represented by bold traces. The trajectory of mMCT1/mBSG marked by a red arrow in **h** and **i** corresponds to the same one in Fig. 6e, f. **i** Changes in the angles of TM0 in mBSG, TM3 and TM6 in the NTD of mMCT1 relative to the membrane surface over a representative trajectory. **j** Boxplots showing H-bonds between mBSG’s R207 and mMCT1’s NTD over the trajectories. **k** The binding affinities of 6E7F1 to both WT mMCT1/mBSG and the mMCT1/mBSG (K111A/K127A) variant measured by Octet biolayer interferometry. Purified 6E7F1 was immobilized on Anti-Mouse IgG Fc Capture biosensors. **l** The transport activities of mMCT1, WT mMCT1/mBSG, and mMCT1/mBSG (K111A/K127A) assessed in the absence or presence of 6E7F1.

To understand the impact of 6E7F1 on mMCT1/mBSG transport activity, we investigated mBSG’s structural dynamics in both mMCT1/mBSG and mMCT1/mBSG/6E7F1Fab complexes. We observed significant H-bond interactions between mBSG and 6E7F1Fab in MD trajectories (Supplementary Fig. S16a), and an increase in β-strands in mBSG upon 6E7F1Fab binding (Fig. 7a). Specifically, I99–G119 segment, which includes strands A and A′, exhibited enhanced H-bond interactions between strands A and B, as well as between strands A′ and G (Fig. 7b–f). MD trajectories also revealed more H-bonds between strand A′ (specifically residues S116 and E118) and the anchor residue R207 of mBSG (Supplementary Fig. S16b–d). Additionally, the root mean square fluctuation (RMSF) values for the Cα atom of each residue within the Ig2 domain were lower in mMCT1/mBSG/6E7F1Fab (Fig. 7g). Rotational motility of the Ig2 domain was absent in all simulations (Fig. 7h; Supplementary Video S2), suggesting that 6E7F1Fab binding reduced the conformational flexibility of mBSG’s Ig2 domain.

Our aforementioned simulation and functional analyses indicate that H-bond interactions between TM0 of mBSG and the NTD of mMCT1 are crucial for domain movement. 6E7F1Fab binding to mBSG reduced the extracellular H-bonds between TM0 and NTD (Fig. 7j; Supplementary Fig. S16b, c, e). The combined reduction in Ig2 domain flexibility and TM0–NTD binding affects the conformational dynamics of TM0 and NTD in the mMCT1/mBSG/6E7F1Fab complex. Consequently, TM0 and NTD did not transition from an outward to an inward conformation in simulations (Fig. 7i; Supplementary Fig. S17).

To validate our MD simulations, we sought to create an mBSG variant that counters 6E7F1’s ‘freezing’ effect on the Ig2 domain. The mBSG (K111A/K127A) variant achieved this by exhibiting fewer β-strands in the Ig2 domain compared to the WT mBSG when bound by 6E7F1Fab (Supplementary Fig. S15b–d). Consistent with this result, the mMCT1/mBSG (K111A/K127A) variant showed approximately double the transport activity of the WT mMCT1/mBSG upon 6E7F1 binding (Fig. 7l). Additionally, the mMCT1/mBSG (K111A/K127A) variant had a similar binding affinity for 6E7F1 to the WT mMCT1/mBSG (Fig. 7k; Supplementary Fig. S7c, d), indicating that its increased transport activity was not due to weaker antibody affinity. Despite this, the Ig2 domain of the mMCT1/mBSG (K111A/K127A) variant still increases β-strand proportions upon 6E7F1 binding (Supplementary Fig. S15c, d). Additionally, antibody binding impairs both extracellular and intracellular H-bonds between mBSG’s TM0 and mMCT1’s NTD (Supplementary Fig. S15e, f). As expected, the transport activity of mMCT1/mBSG (K111A/K127A) decreases in the presence of 6E7F1 (Fig. 7l). Collectively, 6E7F1 modulates mMCT1’s transition rate by decreasing mBSG’s Ig2 flexibility and disrupting interactions between mBSG and mMCT1.

### Concluding remarks

Targeting both MCT1 and MCT4, along with BSG, could provide a powerful strategy against various tumors^4,10,11,18,19^. In this study, we demonstrate that an anti-BSG antibody can meet these criteria by acting as a dual inhibitor of MCT1 and MCT4 through its interaction with BSG. Mechanistically, the antibody reduces the MCT transition rate by decreasing the flexibility of BSG’s Ig2 domain and weakening the interactions between BSG’s TM and MCT. Other researchers have also observed that disrupting the disulfide bridge within the Ig2 domain of BSG impairs the transport activity of MCT1 and MCT4^17,53^. These findings suggest that the Ig2 and TM domains of BSG are promising targets for the development of pan-MCT modulators in the future. Based on our understanding of antibody inhibition mechanisms, we can optimize affinity by retaining known key interactions and augmenting additional ones. Higher specificity is expected to reduce off-target effects and potential drug resistance, leading to more effective therapeutic outcomes.

The 6E7F1 antibody may have promising potential in treating tumors with high BSG expression, such as adrenocortical carcinoma (ACC), HCC, lung adenocarcinoma (LUAD), pancreatic adenocarcinoma (PAAD), cholangio carcinoma (CHOL), kidney renal clear cell carcinoma (KICH), esophagealcarcinoma (ESCA), and skin cutaneous melanoma (SKCM)^13,26,54,55^. The correlation between BSG and prognosis in these tumors is crucial for its clinical application. However, since BSG is also highly expressed in normal tissues like the testis, skeletal muscle, placenta, heart, colon, proximal tubular cells^13,55^, off-target effects must be considered.

Our research demonstrated that the combination of anti-BSG and anti-PD-1 antibodies has synergistic effects in cancer immunotherapy. Other studies suggest that inhibiting tumor glycolysis alongside CTLA-4 blockade could enhance effector T cell immunity by destabilizing Treg cells within the TME^29,35,36,56^. Consequently, bispecific antibodies targeting both BSG and either PD-1, PD-L1, or CTLA-4 hold promise for improving patient outcomes.

The metabolic changes observed in NSCLC-PDOs indicate significant adaptations in both PDOs and immune cells when exposed to anti-PD-1 antibody alone or in combination with BSG antibodies. Metabolic profiling of PDOs and tumor-infiltrating immune cells can reveal patient-specific vulnerabilities, guiding personalized therapeutic strategies, such as combination therapies with immune-based or targeted drugs. Integrating PDO metabolic profiling with in vivo models is crucial for validating these therapeutic approaches, bridging the gap between preclinical findings and clinical applications, and fostering innovation in cancer treatment.

## Materials and methods

### Protein expression and purification

#### Expression and purification of mMCTs, mMCTs/mBSGs, and their variants

The coding sequences for WT mMCT1, mMCT4, mBSG, and their mutational or truncational variants were each cloned into pEG BacMam vector, a gift from Eric Gouaux’s lab^57^. Unless stated otherwise, 8×His-Flag tags were placed at the C-termini of proteins and connected by a Rhinovirus 3C protease-cleavable linker. The proteins were expressed in HEK293S GnTI^−^ (N-acetylglucosaminyl-transferase I-negative) cells (ATCC, #CRL-3022)^58^. Cells were collected by centrifugation (5000× *g*, 10 min, 4 °C) and disrupted by KIMBLE Dounce tissue grinder (Merck Millipore) in a buffer containing 50 mM HEPES, pH 7.5 and 150 mM NaCl. The buffer was supplemented with 5.2 µg/mL aprotinin, 2 µg/mL leupeptin, and 1.4 µg/mL pepstatin A (all from Merck Millipore). Cell debris was removed by centrifugation (10,000× *g*, 25 min, 4 °C). The membrane fraction was collected by ultracentrifugation (100,000× *g*, 1 h, 4 °C), and solubilized for 2.5 h at 4 °C in a buffer containing 50 mM HEPES, pH 7.5, 150 mM NaCl, 1% (w/v) n-dodecyl-D-maltoside (DDM, Anatrace), and 0.1% (w/v) cholesteryl hemisuccinate (CHS, Anatrace). Insoluble materials were removed by ultracentrifugation (100,000× *g*, 1 h, 4 °C). The detergent-soluble fraction was incubated with Ni-NTA resin (Qiagen), and incubated for 2 h at 4 °C. The beads were eluted with an elution buffer containing 20 mM HEPES, pH 7.5, 150 mM NaCl, 0.03% DDM, 0.003% CHS, and 250 mM imidazole, and further incubated for 2 h with anti-DYKDDDDK G1 Affinity Resin (GenScript) to improve protein purity. The protein sample was then eluted by DYKDDDDK peptide (GenScript) and purified by size-exclusion chromatography (SEC) on a Superdex 200 Increase 10/300 GL column (GE Healthcare), equilibrated with a SEC buffer containing 20 mM HEPES, pH 7.5, 150 mM NaCl, 0.03% DDM, and 0.003% CHS. The peak fractions of the protein sample were collected and concentrated to 2.0–5.0 mg/mL, using a 100 kDa MWCO Amicon centrifugal filter (Merck Millipore). Each protein or protein complex was flash frozen and stored in liquid nitrogen for further usage.

#### Expression and purification of Ig1 and Ig2 domains of mBSG

The coding sequences for Ig1 and Ig2 domains were each cloned into the pET28a vector (Addgene) between the *Nde*I and *Xho*I restriction sites. The proteins were each expressed in *E. coli* BL21 (DE3) cells. Cells were harvested and resuspended in buffer containing 50 mM HEPES, pH 7.5, and 100 mM NaCl, and then lysed via homogenizer. Ig1- or Ig2-containing supernatant was incubated with Ni-NTA resin for 3 h at 4 °C. The beads were washed with a buffer containing 20 mM HEPES, pH 7.5, 100 mM NaCl, and 30 mM imidazole, and then eluted with a buffer containing 20 mM HEPES, pH 7.5, 100 mM NaCl, and 300 mM imidazole. The protein was concentrated and purified by SEC on a Superdex 200 Increase 10/300 GL column equilibrated with a buffer containing 20 mM HEPES, pH 7.5 and 150 mM NaCl.

#### Expression and purification of saposin A

Saposin A with an N-terminal 6×His-tag and thrombin cleavage site in a pNIC28-Bsa4 vector was expressed in the *E.* *coli* strain Rosetta-gami B(DE3)^59^. The Rosetta-gami B(DE3) cells were harvested, resuspended and lysed in a buffer containing 50 mM HEPES, pH 7.5, 300 mM NaCl and 1% (w/v) Triton X-100 (Merck Millipore). The cell lysate was heated at 85 °C for 10 min to precipitate all thermolabile components. Saposin A-containing supernatant was incubated with Ni-NTA resin for 3 h at 4 °C. The beads were washed with a buffer containing 20 mM HEPES, pH 7.5, 300 mM NaCl, 50 mM sodium cholate, and 30 mM imidazole. The saposin A was eluted with a buffer containing 20 mm HEPES, pH 7.5, 300 mm NaCl, 50 mM sodium cholate, and 300 mM imidazole. The protein elute was incubated with thrombin (Merck Millipore) to get rid of the 6×His-tag and dialyzed overnight at 4 °C against a dialysis buffer containing 20 mM HEPES, pH 7.5, 300 mM NaCl, and 5% (w/v) glycerol. The cleaved protein was concentrated and purified by SEC on a Superdex 200 Increase 10/300 GL column equilibrated with a SEC buffer containing 20 mM HEPES, pH 7.5 and 150 mM NaCl.

#### Reconstitution of WT mMCT1/mBSG into Salipro nanoparticles

We reconstituted WT mMCT1/mBSG into Salipro nanoparticle following an established protocol^59^. Briefly, the 10 mM lipid stock solution containing a mixture of POPC/POPE/POPG lipids (in a 3:1:1 ratio, all from Avanti) was prepared. mMCT1/mBSG was then mixed with saposin A and POPC/POPE/POPG lipid mixture in a molar ratio of 1:8:10 using a buffer containing 20 mM HEPES, pH 7.5, and 150 mM NaCl. The mix was incubated for 2 h at 4 °C. The Salipro formation was initiated by adding 160 mg/mL equilibrated SM2 biobeads (Bio-Rad) to remove detergents. The mixture was agitated overnight at 4 °C. The sample was additionally incubated for 30 min at 4 °C with 160 mg/mL of equilibrated SM2 biobeads twice to remove residual detergents. The sample was filtered through a 0.22-μm filter and subjected to SEC using a Superose 6 Increase 10/300 column (GE Healthcare), which was equilibrated with a buffer containing 20 mM HEPES, pH 7.5, and 150 mM NaCl. SEC peak fraction was pooled and concentrated to 2.0 mg/mL. The sample was stored at –80 °C for further usage.

### mBSG antibody discovery and production

#### Screening of mBSG antibodies that inhibit the transport activities of mMCT1 and mMCT4

C57BL/6 mice were immunized with 50 μg of mBSG Ig1 or Ig2 antigens, followed by four boosts of 50 μg antigen each. Hybridoma cells (~2000 per antigen) were generated by fusing SP2/0-Ag14 mouse myeloma cells with splenic B lymphocytes from the immunized animals. Culture supernatants were screened using ELISA in detergent-free conditions. The ELISA-positive supernatants were further analyzed in fluorescence-based flux assays using HPTS-encapsulated liposomes to identify polyclonal antibodies inhibiting mMCT1/mBSG-mediated pyruvate influx and mMCT4/mBSG-mediated L-lactate influx. Anti-Ig2 polyclonal antibodies were found to be effective at inhibiting transporter activities. Subsequently, monoclonal antibodies from ~10 hybridomas were purified using affinity chromatography.

#### Antibody sequencing

RNA was extracted from hybridoma cells using HiPure RNA Mini Columns (Magen) following the manufacturer’s instructions. cDNA was synthesized from the RNA using SMARTScribe Reverse Transcriptase (Takara) with oligo-dT and template switch oligo (TSO). The resulting cDNA was diluted for amplification. In the first-stage PCR, heavy-chain and light-chain fragments were separately amplified using primers anchored to the TSO and constant regions. Magnetic beads were used to purify PCR products. In the second-stage PCR, index primers were added to create a TruSeq dual index library. The libraries were quantified and sequenced on an Illumina MiSeq platform. Raw fastq files underwent quality assessment, and poor-quality adapters and bases were removed. Merged sequences were processed using IgBLAST software to identify V(D)J sequences, with reference sequences obtained from the IMGT database^60^.

#### The preparation of 6E7F1Fab fragment

The 6E7F1 antibody (IgG2 subclass) was chosen for its potent inhibitory effects on both mMCT1 and mMCT4 transporter activities. To create 6E7F1Fab fragment, the full-length antibody underwent digestion with papain (1:100 w/w) for 5 h at 37 °C in a buffer containing 20 mM NaPO_4_, pH 7.5, 150 mM NaCl, 1 mM EDTA, and 10 mM L-cysteine hydrochloride. After digestion, papain was inactivated with 30 mM iodoacetamide on ice for 15 min. The resulting digested Fab fragment was purified using SEC on a Superdex 200 Increase 10/300 column equilibrated with a buffer containing 20 mM HEPES, pH 7.5 and 150 mM NaCl. The purified Fab was stored at –80 °C for future use.

### Fluorescence-based liposomal flux assay

#### Preparation of HPTS-encapsulated fluorescent proteoliposomes

A lipid mixture containing POPC, POPE, POPG, and cholesterol (all from Avanti) at a 3:1:1:1 ratio (w/w) was dried under an argon stream. Dried lipids (10 mg/mL) were resuspended in a buffer (20 mM Tris, pH 8.5, 150 mM NaCl) using sonication and incubated with n-Octyl-β-D-Maltopyranoside (OM, Anatrace) for 2 h at room temperature. Each purified protein sample of mMCT1, mMCT4, mMCT1/mBSG, mMCT4/mBSG, or their variants was added to the lipid/OM mixture at a protein-to-lipid molar ratio of 1:1000. After 4-h incubation at room temperature, SM2 biobeads (Bio-Rad) were used to remove detergents by overnight incubation at 4 °C. The mixture was subsequently incubated with SM2 biobeads for an additional 30 min at 4 °C to remove residual detergents. Liposome fractions that could be stained with Bradford dye (Bio-Rad), were collected and concentrated using an Amicon Ultra centrifugal filter (0.5 mL, 100 kDa cutoff) to achieve a final concentration of 2.5 mg/mL. The protein concentration after reconstitution was measured in the same way as described above, and further confirmed by Coomassie blue staining and western blot.

To load the pH-sensitive fluorescent dye HPTS (Merck Millipore), proteoliposomes were mixed with 0.1 mM HPTS and sonicated in an iced water bath to prevent overheating. The mixture was then extruded 25 times through a polycarbonate filter (0.2 μm pore size) using a lipid extruder (Avanti). After extrusion, any untrapped dye was removed by passing the mixture through a Sephadex G-50 column, eluted with a buffer containing 20 mM Tris, pH 8.5 and 150 mM NaCl. The resulting eluted fraction containing HPTS-encapsulated proteoliposomes was collected and concentrated to either 2.0 mg/mL or 2.5 mg/mL. Additionally, protein-free liposomes (designated as ‘empty’ liposomes) were prepared in parallel using the same method but without protein. Freshly prepared HPTS-encapsulated proteoliposomes and empty liposomes were used for fluorescence-based flux assays within 48 h of preparation, with assays executed at 25 °C. Unless otherwise specified, HPTS-encapsulated empty liposomes served as the negative control, following the same procedure as the proteoliposomes.

#### Substrate selectivity assay

The assays were executed with a SpectraMax i3 (Molecular Devices) plate reader based on a modified version of a previously published method^28^. In each well of a Nunc MicroWell 96-well plate (Thermo Fisher Scientific), 85 μL of extraliposomal buffer (20 mM HEPES, pH 7.0, 150 mM NaCl) was added, followed by gentle mixing with 10 μL of HPTS-encapsulated proteoliposomes. After a 10-min incubation, baseline fluorescence F_*start*_ was recorded every 15 s for 8 cycles at excitation and emission wavelengths of 450 nm and 510 nm, respectively. Then 5 μL of test anions (each at a final concentration of 25 mM sodium salt and pH 7.0) was added to initiate H^+^ influx, and the fluorescence signal (F) was monitored every 15 s for 60 cycles. Following 15 min of flux signal monitoring, the proteoliposomes were sonicated to allow H^+^ and test anions to pass the membrane and reach equilibrium. The final fluorescence value F_*end*_ was then recorded.

#### H^+^/pyruvate influx under pyruvate gradients or H^+^/L-lactate influx under L-lactate gradients

We assessed the impact of pyruvate gradient on H^+^/pyruvate influx mediated by mMCT1 or mMCT1/mBSG. The assay followed the substrate selectivity protocol with the following adjustments: after recording the baseline fluorescence F_*start*_, we initiated H^+^/pyruvate influx using pyruvate at pH 7.0, with final concentrations of 0 mM, 5 mM, 25 mM, or 100 mM. Similarly, to examine the influence of an L-lactate gradient on H^+^/L-lactate influx mediated by mMCT4 or mMCT4/mBSG, we performed a similar assay, replacing pyruvate (pH 7.0) with L-lactate (pH 7.0).

#### H^+^/pyruvate influx at H^+^ gradients

We assessed the impact of H^+^ gradient on H^+^/pyruvate influx mediated by mMCT1 or mMCT1/mBSG. The assay followed the substrate selectivity protocol with the following adjustments: we used extraliposomal buffers (20 mM Tris, pH 8.5, HEPES, pH 7.5 or 7.0, and MES, pH 6.5) with 150 mM NaCl; H^+^/pyruvate influx was initiated using 25 mM pyruvate at the specified pH values after recording of F_*start*_.

#### H^+^/pyruvate influx mediated by mMCT1/mBSG mutational and truncated variants

The assay followed the substrate selectivity protocol with the following adjustments: influx was initiated using 25 mM pyruvate at pH 7.0 after recording the initial fluorescence F_*start*_, the empty liposomes and proteoliposomes containing WT mMCT1 or WT mMCT1/mBSG were tested in parallel as controls.

#### H^+^/pyruvate influx or H^+^/L-lactate influx inhibited by 6E7F1 and/or AZD3965

HPTS-encapsulated proteoliposomes containing each protein (WT mMCT1, WT mMCT4, WT mMCT1/mBSG, WT mMCT4/mBSG, or their variants, each at a final concentration of 2.0 mg/mL) were mixed with 0.6 μM 6E7F1, 0.6 μM AZD3965, 0.3 μM 6E7F1 + 0.3 μM AZD3965, or 0.6 μM 6E7F1 + 0.6 μM AZD3965 in a buffer (20 mM Tris, pH 8.5 and 150 mM NaCl) for 2 h at 4 °C with gentle rotation. After incubation, 10 μL of each mixture was combined with 85 μL of extraliposomal buffer for fluorescence-based flux assays. IgG and/or 0.03% DMSO served as controls for 6E7F1 and/or AZD3965.

### In vitro and in vivo tumor models

#### Tumor cell culture

A549 (ATCC, #CCL-185), A20 (ATCC, #TIB-208), and B16F10 (ATCC, #CCL-6475) cells were cultivated in RPMI 1640 (Merck Millipore) with 10% fetal bovine serum (FBS, Thermo Fisher Scientific), penicillin (100 U/mL, Merck Millipore) and streptomycin (100 μg/mL, Merck Millipore). A549 and B16F10 cells can be cultured under normoxic (21% O_2_, 5% CO_2_ and 37 °C) and hypoxic (1% O_2_, 5% CO_2_ and 37 °C) conditions. In contrast, A20 cells can only survive normoxic condition.

#### Tumor cell viability assay

Cells were seeded in 96-well culture dishes in media (For A20 cells, 3000 cells per well; for A549 cells and B16F10 cells, 1500 cells per well). A20 cells were seeded and treated with the indicated concentrations of AZD3965 and/or 6E7F1 in complete medium for 72 h. IgG and DMSO group were set as controls. Then cell viability was determined with CCK8 kit in SpectraMax i3 (Molecular Devices). For A549 cells and B16F10 cells, cells were incubated overnight to adhere and were treated as indicated under normoxic or hypoxic conditions for 72 h before cell viability was measured.

#### Measurement of lactate concentration

A20 cells were seeded and treated with the indicated concentrations of AZD3965 and/or 6E7F1 in complete medium for 72 h. IgG and DMSO group were set as controls. The lactate concentration was measured with Lactate Colorimetric/Fluorometric Assay Kit (BioVision, Cat# K607-100) in SpectraMax i3 (Molecular Devices). For A549 cells and B16F10 cells, cells were incubated overnight to adhere and were treated as indicated under normoxic or hypoxic conditions for 72 h before lactate concentration was determined.

#### siRNA transfection

The following siRNA sequences are used for cell transfection: siNT (UAGCGACUAAACACAUCAA), siBSG (GGUCAGAGCUACACAUUGA), siMCT1 (ACUGCUGUGUUGCUGGAGC), siMCT4 (CGACCCACGUCUACAUGUACGUGUU). Lipofectamine RNAiMax (Thermo Fisher Scientific) was used for siRNA transfection, according to the manufacturer’s instructions.

#### Immunofluorescence staining

The treated cells were cultured on small round cover glasses, fixed with 4% paraformaldehyde, permeabilized with 0.2% Triton X-100, blocked with 5% bovine serum, and treated with mouse anti-EMMPRIN/CD147 antibody (Santa Cruz Biotechnology), and rabbit anti-MCT4 or MCT1 antibody (Thermo Fisher Scientific) overnight at 4 °C. After incubation with the corresponding fluorescence-labeled secondary antibodies (Alexa Fluor 555-labeled goat anti-mouse IgG (H + L), and Alexa Fluor 488-labeled goat anti-rabbit IgG (H + L); both from Thermo Fisher Scientific), the samples were visualized under an OLYMPUS IX83 confocal florescence microscope to assess co-localization of BSG and MCT1 or MCT4.

#### Allograft and xenograft models

Animal experiments were conducted in accordance with approved protocol guidelines of the Animal Experimentation Ethics Committee of the Guangxi Medical University Cancer Hospital (LW2022061). Three- to four-week-old male or female BALB/c nude mice and C57BL/6 mice were purchased from Animal Center of Guangxi Medical University. To establish A549 xenograft and B16F10 allograft mouse models, 1 × 10^6^ A549 or B16F10 cells in 100 μL of serum-free RPMI 1640 medium were injected subcutaneously into the flank region of a BALB/c nude mouse and a immunocompetent C57BL/6 mouse, respectively. For A549 xenograft model, mice were randomly assigned to treatment or control groups of six animals each. The mice were treated intraperitoneally with 6E7F1 antibody (5 mg/kg) every other day. AZD3965 (50 mg/kg) was administered twice daily by oral gavage on the same day of 6E7F1. Mice were treated by either 6E7F1 or AZD3965 or their combination for a total of seven times. The tumor size was measured during drug administration and this measurement was continued for up to an additional two weeks before mice were sacrificed. Tumor size was measured using a digital caliper and the volume was calculated using the formula: Volume (mm^3^) = (length × width^2^)/2. For the B16F10 allograft model, C57BL/6 mice were randomly assigned to treatment or control groups of five animals each. 6E7F1 or AZD3965 was administered in the same ways as for the A549 model. The anti-mouse PD-1 antibody (10 mg/kg, BioXcell, BE0146) was intraperitoneally injected into mice alone, or in combination with 6E7F1 or AZD3965. After drugs were administered seven times, mice were sacrificed and tumors were dissociated to single cells for FACS analysis.

### Human subject

This study was approved by the Ethics Committee of Tianjin Cancer Hospital and the ethics number is bc20240034. All patients were given informed consent for sample collection. Four patients pathologically diagnosed with NSCLC at Tianjin Cancer Hospital were enrolled for organoid construction.

### Organoid culture

#### Organoid construction

Tumor samples from NSCLC patients were collected and washed three times with Advanced DMEM/F12 medium containing 2% penicillin-streptomycin, each time for 5 min. These tissue blocks were cut into ~1 mm^3^ fragments and incubated with digestion buffer (DMEM/F12 with 1% penicillin/streptomycin, 0.1 mg/mL gentamicin, 0.6% nystatin, 1 mg/mL collagenase A, 0.096 mg/mL hyaluronidase) at 37 °C for 30 min on a shaker. Digestion was terminated with PBS buffer, and the digested solution was filtered through 70-μm cell strainers. Cells were then seeded at 2–5 × 10^4^ cells/10 μL Matrigel/well in 48-well plates. After the solidification of Matrigel at 37 °C in a CO_2_ incubator for ~10 min, the organoid culture medium was added for further culture under normoxia (21% O_2_) or hypoxia (1% O_2_) and was refreshed at an interval of 3–5 days (T2310-LC100, OneTar Biomedicine).

#### T cell expansion and maintenance

Human peripheral blood mononuclear cells (PBMCs) were isolated from whole blood (~5 mL) collected in Vacutainer containing sodium heparin (BD Vacutainer, Franklin Lakes, NJ, USA) from patients by centrifuging at 1500× *g* for 20 min. The interface cells were harvested and washed twice with PBS (5% fetal calf serum) at 800× *g* and 500× *g* for 10 min, respectively. PBMCs were resuspended and cultured in complete RPMI 1640 medium containing 10% pooled human serum (PHS; Gemini Bio-Products, Woodland, CA, USA), 1% penicillin/streptomycin (MediaTech, Herndon, VA, USA), 50 μM β-mercaptoethanol (Gibco, Invitrogen, Grand Island, NY, USA), and 1% L-glutamine (Gibco, Carlsbad, CA, USA). Sterile recombinant human cytokine IL-2 (150 U/mL, Chiron, CA, USA) was used to support T-cell proliferation.

#### IC_50_ of various drugs on organoids and T cells

Organoids or corresponding T cells were individually seeded at 2000 cells/well in 96-well culture plates, followed by treating with the indicated reagents after 2–3 days at 37 °C in a low-oxygen incubator (1% O_2_) until the organoid size reached ~50 µm (AZD3965 (MCT1 inhibitor), VB124 (MCT4 inhibitor), and GSK2837808A (LDHA inhibitor): 0.01–100 µM (10-fold dilution); 6E7F1: 0.0006–0.6 µM (10-fold dilution)). After 72-h incubation, optical images were captured. Cell viability was assessed using the CellTiter-Glo™ reagent (Promega, G9683). The reagent was added to the wells containing organoids and T cells, followed by incubation in the dark on a shaker for 30 min. Luminescence was measured using a microplate reader.

#### Co-culture of organoids and T cells under hypoxic conditions

Organoid cells and corresponding T cells were mixed at a ratio of 1:5, and cultured in 96-well culture plates with a total of 12,000 cells/well. The culture was maintained in a low-oxygen incubator (1% O_2_) for 48 h. After 10 h of co-culture, the cell mixtures were analyzed using CTG luminescence, along with live/dead fluorescence staining. Inter-group CTG luminescence values were compared and analyzed. Tumor organoids were pre-stimulated with IFNγ (200 ng/mL) to enhance antigen presentation. The culture medium included IL-2 to support T-cell proliferation. The anti-CD3/CD28 antibody (5 µg/mL) was plate-bound to provide co-stimulation. T-cell reactivity was assessed by evaluating CD107a expression in the presence or absence of tumor organoids. The reagent concentrations for the assay were determined based on their IC_50_ values, ensuring safety for T cells. Pembrolizumab (a PD-1 antibody by Merck & Co.) was administered to NSCLC-PDOs at a concentration of 48 µg/mL^61,62^, either alone or in combination with other reagents, with or without T cell co-culture.

#### Flow cytometry

Tumor tissues from various treatment groups were digested into single-cell suspensions (2–3 × 10^5^ cells per sample), which were then washed with PBS buffer including 0.5% (w/v) BSA and incubated with antibodies for staining at 4 °C for 30 min. After washing, the cells were suspended in 150 μL of staining buffer and subjected to analysis by the Attune Nxt flow cytometer (Thermo Fisher Scientific) with 2–3 × 10^4^ cells recorded per sample. Antibodies used for this study are as below: CD3-APC (BioLegend, clone 17A2), CD4-AF488 (BioLegend, clone Gk1.5), CD11c-AF700 (BioLegend, clone N418), CD45-Percp (BioLegend, clone 3.0-F1), Ly-6G-APC-Cy7 (BioLegend, clone EA8), NK-1.1-BV650 (BioLegend, clone PK136), CD8-PE (BD, clone 53-6.7), PD-1-BV421 (BioLegend, clone J43), F4/80-PE-Cy5 (BioLegend, clone BM8), CD107a-BV711 (BioLegend, clone H4A3), CD19-BV711 (BioLegend, clone 6D5), CD11b-PE-Cy7 (BD, clone M1/70), CD25-BV510 (BD, clone 3C7), Fc block (1:100) (BD).

#### HE and IHC staining

Tumor tissues were fixed in 4% paraformaldehyde, followed by dehydration and generation of paraffin-embedded blocks. Organoids were taken from culture plates and embedded in agarose gel, fixed in 4% paraformaldehyde, dehydrated, and embedded in paraffin for blocks. These tissue and organoid-derived paraffin sections were subjected to HE and IHC staining. Antibodies used for this study included: TCK7 (MXB, Kit-0021), TTF-1 (MXB, MAB-0677), NapsinA (MXB, MAB-0704), EpCAM (MXB, MAB-0850).

#### scRNA-seq

scRNA-seq libraries were generated using the BD Rhapsody WTA Amplification Kit (#633801). The sequencing data was analyzed using the Rhapsody Sequence Analysis Pipeline (v2.0) with default parameters, and mapped by the RhapRef_Human_WTA_2023-02 transcriptome. Quality control, dimensionality reduction, and clustering were conducted using Seurat (v4.0.3)^63^. For each sample, cells expressing at least 200 genes and up to 7500 genes were considered high quality. Criteria for retention included: < 20% mitochondrial genes and < 5% red blood cell genes; genes expressed in at least 3 cells. Doublet cells were excluded using DoubletFinder (v2.0.2)^64^. Harmony was utilized to correct batch effects^65^. Principal components (PCs) obtained from RunPCA were used in RunUMAP and FindNeighbors. Clusters were identified using FindClusters with a resolution of 0.6. Differentially expressed genes within each cluster were identified using the Wilcoxon Rank Sum test via FindAllMarkers. Clusters were annotated based on classical marker genes and differentially expressed genes. Single-cell metabolic activity was quantified using scMetabolism (v0.2.1)^66^.

### Cryo-EM and MD simulations

#### Grid preparation and data acquisition

For cryo-EM structure determination, WT mMCT1/mBSG/6E7F1Fab reconstituted into Salipro nanoparticles was freshly made. 2.3 μL purified protein complex at concentration of 0.7 mg/mL were applied to glow-discharged holy carbon grids (Quantifoil R1.2/1.3 Cu 300 mesh). Excess liquid was removed in Vitrobot Mark IV (Thermo Fisher Scientific) under 10 °C and 100% humidity conditions, by blotting grids for 3.5–4.5 s (with a blotting force of –1). Grids were subsequently flash-frozen in liquid ethane.

Two datasets (“dataset 1” and “dataset 2”) were collected on two different 300 kV Titan Krios (FEI), each equipped with a K2 Summit direct detector and a GIF Quantum energy filter at 165,000× magnification. Movie stacks were automatically acquired using SerialEM^67^ with a 20 eV slit width and a defocus range of –1.0 μm to –2.0 μm. Each stack consisting of 48 frames was exposed for 3.004 s with a total dose of ~75 e/Å^2^. Datasets 1 and 2 were recorded in pixel sizes of 0.82 Å/pixel (0.41 Å/pixel in super-resolution) and 0.84 Å/pixel (0.42 Å/pixel in super-resolution), respectively.

#### Cryo-EM image processing

A diagram for data processing is presented in Supplementary Fig. S11. We met similar challenges for the structure determination of mMCT1/mBSG/6E7F1Fab to those for the structure determination of hMCT1/hBSG^29^. The electron micrographs, 2D class averages, and initial model of mMCT1/mBSG/6E7F1Fab all indicated a thick saposin A lipid disc for Salipro nanoparticles. In addition, Ig1 and Ig2 domains in mBSG are flexible. These, together with the small size of TM region of mMCT1, posed a big technical challenge for data processing.

The initial trial that combined noise reduction and re-weighting methods with the “guided multi-reference 3D classification”, which helped the successful reconstruction of the atomic model of hMCT1/hBSG^29^, did not successfully eliminate the strong noises of saposin A lipid disc. Nevertheless, the guided multi-reference 3D classification^68^ was found to be effective in identifying good particles for model reconstruction. All of the image processing steps were carried out using CryoSPARC 2.15^69^. For dataset 1, movie frames of mMCT1/mBSG/6E7F1Fab micrographs were motion-corrected and binned two-fold, and dose-weighted using Patch motion. CTF correction was performed using Patch CTF. With a box size of 256 pixels, 1,610,675 particles were automatically picked from 5895 micrographs. Two rounds of 2D classification gave rise to 620,207 particles that showed good secondary structural features of the entire protein complex including the TM region. A good reference of low resolution, and three bad references were generated by 3D classification, and then applied to guided multi-reference classification. Four rounds (three parallel runs each round) of guided multi-reference 3D classification were performed using the heterogeneous refinement job. Particles from the best classes were merged and duplicated particles were removed. 339,596 particles were selected for further ab initio reconstruction and heterogeneous refinement, which generated two good references, wherein one showed features for the TM regions and part of extracellular regions and the other showed features for extracellular regions of the protein complex. These two good references and a bad reference were used for additional five rounds (three parallel runs each round) of guided multi-reference classification. Particles from the best classes were merged and duplicated particles were removed, resulting in a dataset of 129,990 particles. Further homogeneous refinement and non-uniform refinement generated a model consisting mainly of single-pass TM of mBSG and TM region of mMCT1, which displayed clear secondary structural features at an average resolution of 5.03 Å.

Because the quality of the density map generated by dataset 1 was not enough to reveal atomic details of 6E7F1Fab and extracellular region of mBSG, we collected dataset 2 for the complex. The data processing procedure was similar to that of dataset 1. 712,923 particles were automatically picked from 2842 micrographs. After 2 rounds of 2D classification, 379,840 particles were selected for ab initio reconstruction, which generated two good references, wherein one showed features for the saposin A lipid disc and the other showed features of the extracellular region of the protein complex. These two good references and a bad reference were used for four rounds (three parallel runs each round) of guided multi-reference classification. Particles from the best classes were merged and duplicated particles were removed, resulting in a dataset of 106,160 particles. Further homogeneous refinement and non-uniform refinement generated a model consisting mainly of Ig2 domain of mBSG and 6E7F1Fab at an average resolution of 3.23 Å. However, the refinements did not improve the density for TM regions of mBSG and mMCT1.

In all cases, resolution was estimated in the presence of a soft solvent mask and based on the gold standard Fourier shell correlation (FSC) 0.143 criterion^70,71^. Local resolution was estimated using default parameters. Only the map for 6E7F1Fab and extracellular region of mBSG was sharpened with B factors estimated in the non-uniform refinement and was low-pass filtered at its resolution.

#### Model building, refinement, and validation

The atomic coordinates of 6E7F1Fab and the extracellular region of mBSG were generated by combining homology modeling and de novo model building. An initial structure model of 6E7F1Fab was firstly predicted by AlphaFold^72^. The template used for the homology modeling of extracellular region of mBSG was the crystal structure of hBSG^51^ (PDB: 3B5H). The model was manually adjusted in COOT^73^ according to the cryo-EM map. The improved model of mBSG/6E7F1Fab complex was refined against the corresponding map using PHENIX^74^ in real space with secondary structure and geometry restraints. Overfitting of model was monitored by refining the model in one of the two independent maps from the gold-standard refinement approach, and testing the refined model against the other map^75^. Model geometries were assessed using Molprobity as part of the PHENIX validation tools.

The density of the TM regions for both mBSG and mMCT1 was not enough for atomic model building. Cryo-EM structure of TM regions of hBSG and hMCT1 in the presence of lactate^50^ (PDB: 6LZ0) was used and rigid-body fitted into the density in Chimera^76^ with good agreement. To reduce ambiguity in model building, all side chains were truncated to C_ß_. Thus, the overall structure of mMCT1/mBSG/6E7F1Fab was reconstructed from the 5.0 Å TM region and the 3.2 Å extracellular region. The complex could be rigid-body fitted into ~10.0 Å density in Supplementary Fig. S11m with good agreement.

Cryo-EM data collection, analysis, and refinement statistics are shown in Supplementary Table S1. UCSF Chimera^76^, and PyMOL^77^ were used to prepare structural figures.

#### MD simulations

mMCT1 and hMCT1 share > 87% identity in amino acid residues, and mBSG and hBSG share > 57% identity (Supplementary Figs. S1 andS2). The homology models of outward-facing mMCT1, the TM region of outward-facing mMCT1/mBSG, and the TM region of outward-facing mMCT1/mBSG/6E7F1Fab, were built using the TM region of outward-facing hMCT1/hBSG (PDB: 6LZ0)^50^. The homology model of inward-facing mMCT1/mBSG was built using the inward-facing hMCT1/hBSG (PDB: 7CKO). The TM region of each structure was inserted into a lipid bilayer containing 180 units of POPC and 45 cholesterols in each leaflet using CHARMM-GUI membrane builder^78,79^. Each complex was solvated in TIP3P water molecules^80^ with 150 mM KCl.

All MD simulations were performed using AMBER 20 package. The parameters for proteins and membranes were obtained from AMBER ff19SB^81^ and lipid17 force fields^82,83^, respectively. Hydrogen mass repartition^84,85^ was used for all simulations, allowing for production simulations using a time step of 4 fs. Long range electrostatics was treated by the particle-mesh Ewald (PME)^86^ method with a 9 Å cutoff in all simulations. The systems were maintained at 303.15 K using the Langevin dynamics with a coupling constant of 1 ps^−1^. The SHAKE/RATTLE^87,88^ was used to constrain covalent bonds involving hydrogen atoms to their experimental lengths, and the SETTLE algorithm^89^ was utilized for water. In each trajectory, the initial structure of complex was first subjected to minimization and pre-equilibration (325 ps with 1-fs time step followed by 1500 ps with 2-fs time step). A 1200 ns production simulation was performed for each trajectory. For each molecular assembly, three parallel simulations were performed. The VMD program^90^ was employed to analyze and render the MD trajectories, as well as the calculation of RMSF for Cα atom of each residue, proportion of β-strands and H-bonds. An H-bond is defined if the distance between donor and acceptor is < 3.5 Å and the angle is > 150°. Center-to-center distance between Ig2 domain and mMCT1, namely the distance between the center of all backbone Cα atoms of I99–R207 in mBSG and the center of all Cα atoms of mMCT1, was calculated by TCL scripts within VMD.

### Data analysis

For flux assays, fluorescence data were first normalized to eliminate baseline fluorescence fluctuations using the following equation:

$${F}_{{normalized}}=\frac{{F-F}_{{end}}}{{F}_{{start}}-{F}_{{end}}}$$

Where F_*normalized*_ is the normalized fluorescence plotted in the flux assay figures, ${\rm{F}}$ is measured fluorescence in arbitrary units, F_*start*_ is the average of measured fluorescence before H^+^ influx was initiated by the addition of monocarboxylate substrates, and F_*end*_ is the measured end point fluorescence after disruption of proteoliposomes by sonication. Normalizations were performed with Excel (Microsoft).

Data were plotted from independent biological triplicates unless specified. Prism (GraphPad) was used for data analysis. Statistical significance (*P ≤ 0.05; **P ≤ 0.01; ***P ≤ 0.001; ****P ≤ 0.0001) was determined by Student’s *t*-test. Error bars represent the standard deviation in flux assays and the standard error of the mean in other experiments.

## Supplementary information

Supplementary information

Supplementary Video S1

Supplementary Video S2

## Data Availability

The cryo-EM density maps of mouse MCT1/BSG/6E7F1Fab generated independently from datasets 1 and 2 have been deposited in the Electron Microscopy Data Bank under accession numbers EMD-33559 and EMD-33570, respectively. Coordinates of MCT1/BSG/6E7F1Fab refined against 3.2 Å and 5.0 Å cryo-EM densities, have been deposited in the Protein Data Bank under accession numbers 7Y1B and 7Y1Q, respectively. scRNA-seq data has been uploaded to the Gene Expression Omnibus repository: https://www.ncbi.nlm.nih.gov/geo/query/acc.cgi?&acc=GSE268751. Plasmids and antibodies generated in this study will be available upon request, but we may require a payment and/or a completed Materials Transfer Agreement if there is potential for commercial application.
