# Supplementary material for: A basigin antibody modulates MCTs to impact tumor metabolism and immunity
Source: Cell Discov. 2025 May 6;11:44. doi: 10.1038/s41421-025-00777-1 (PMC12053622; doi:10.1038/s41421-025-00777-1)
Supplement: Supplementary file 1 — Supplementary information [file 41421_2025_777_MOESM1_ESM.pdf]

Supplementary information for

A Basigin Antibody Modulates MCTs to Impact Tumor Metabolism and Immunity

Heng Zhang, [1,*] Xuemei Yang, [1,*] Yue Xue, [2,*], Yi Huang, [3] Yingxi Mo, [3] Yurun Huang, [3] Hong Zhang, [2] Xiaofei Zhang, [5] Weixin Zhao, [5] Bin Jia, [6] , Ningning Li, [7] Ning Gao, [7] Dongxi Xiang,[4,#] Shan Wang, [3,#] Yi Qin Gao, [2,#] Jun Liao [1,8,#]

#, Correspondence: liaojun@shanghaitech.edu.cn; gaoyq@pku.edu.cn; wangshan@sr.gxmu.edu.cn; dxiang@shsmu.edu.cn

The file includes:

Supplementary Figures S1 to S17

Supplementary Table S1

Supplementary Movie titles

Supplementary Fig.S1

```
                                              TM1
mMCT1   --MPPAIGGPVGYTPPDGGWGWAVLVGAFISIGFSYAFPKSITVFFKEIEVIFSATTSEV  58
hMCT1   --MPPAVGGPVGYTPPDGGWGWAVVIGAFISIGFSYAFPKSITVFFKEIEGIFHATTSEV  58
mMCT4   MGGAVVDEGPTGIKAPDGGWGWAVLFGCFIITGFSYAFPKAVSVFFKELMHEFGIGYSDT  60
hMCT4   MGGAVVDEGPTGVKAPDGGWGWAVLFGCFVITGFSYAFPKAVSVFFKELIQEFGIGYSDT  60

                  TM2                       TM3
mMCT1   SWISSIMLAVMYAGGPISSILVNKYGSRPVMIAGGCLSGCGLIAASFCNTVQELYLCIGV  118
hMCT1   SWISSIMLAVMYGGGPISSILVNKYGSRIVMIVGGCLSGCGLIAASFCNTVQQLYVCIGV  118
mMCT4   AWISSILLAMLYGTGPLCSVCVNRFGCRPVMLVGGLFASLGMVAASFCRSIIQIYLTTGV  120
hMCT4   AWISSILLAMLYGTGPLCSVCVNRFGCRPVMLVGGLFASLGMVAASFCRSIIQVYLTTGV  120
                                   ◆

                TM4                         TM5
mMCT1   IGGLGLAFNLNPALTMIGKYFYKKRPLANGLAMAGSPVFLSTLAPLNQAFFDIFDWRGSF  178
hMCT1   IGGLGLAFNLNPALTMIGKYFYKRRPLANGLAMAGSPVFLCTLAPLNQVFFGIFGWRGSF  178
mMCT4   ITGLGLALNFQPSLIMLNRYFNKRRPIANGLAAAGSPVFLCALSPLGQLLQDHYGWRGGF  180
hMCT4   ITGLGLALNFQPSLIMLNRYFSKRRPMANGLAAAGSPVFLCALSPLGQLLQDRYGWRGGF  180
                                                        ●  ●

             TM6
mMCT1   LILGGLLLNCCVAGSLMRPIGPEQVKLEKLKSKESLQEAGKS-------DANTDLIGGSP  231
hMCT1   LILGGLLLNCCVAGALMRPIGPKPTKAGKDKSKASLEKAGKSGVKKDLHDANTDLIGRHP  238
mMCT4   LILGGLLLNCCVCAALMRPLVAPQVGGGTE------------------------------  210
hMCT4   LILGGLLLNCCVCAALMRPLVVTAQPG---------------------------------  207
            ▲        ◆

                                             TM7
mMCT1   KGEKLSVFQTINKFLDLSLFTHRGFLLYLSGNVVMFFGLFTPLVFLSSYGKSKDFSSEKS  291
hMCT1   KQEKRSVFQTINQFLDLTLFTHRGFLLYLSGNVIMFFGLFAPLVFLSSYGKSQHYSSEKS  298
mMCT4   ---PRGPQRPPQRLLDLSVFRDRGFLIYAVAASIMVLGLFVPPVFVVSYAKDMGVPDTKA  267
hMCT4   ----SGPPRPSRRLLDLSVFRDRGFVLYAVAASVMVLGLFVPPVFVVSYAKDLGVPDTKA  263

                TM8                         TM9
mMCT1   AFLLSILAFVDMVARPSMGLAANTKWIRPRIQYFFAASVVANGVCHLLAPLSTTYVGFCV  351
hMCT1   AFLLSILAFVDMVARPSMGLVANTKPIRPRIQYFFAASVVANGVCHMLAPLSTTYVGFCV  358
mMCT4   AFLLTILGFIDIFARPTAGFITGLKKVRPYSVYLFSFAMFFNGFTDLTGSTATDYGGLVV  327
hMCT4   AFLLTILGFIDIFARPAAGFVAGLGKVRPYSVYLFSFSMFFNGLADLAGSTAGDYGGLVV  323

                TM10                         TM11
mMCT1   YAGVFGFAFGWLSSVLFETLMDLIGPQRFSSAVGLVTIVECCPVLLGPPLLGRLNDMYGD  411
hMCT1   YAGFFGFAFGWLSSVLFETLMDLVGPQRFSSAVGLVTIVECCPVLLGPPLLGRLNDMYGD  418
mMCT4   FCIFFGISYGMVGALQFEVLMAIVGTQKFSSAIGLVLLLEAVAVLIGPPSGGKLLDATKV  387
hMCT4   FCIFFGISYGMVGALQFEVLMAIVGTHKFSSAIGLVLLMEAVAVLVGPPSGGKLLDATHV  383

                  TM12
mMCT1   YKYTYWACGVILIIAGIYLFIGMGINYRLLAKEQKAEEK-QKREGKEDEASTDVDEKPKE  470
hMCT1   YKYTYWACGVVLIISGIYLFIGMGINYRLLAKEQKANEQ--KKESKEEETSIDVAGKPNE  476
mMCT4   YKYVFILAGAEVLTSSLVLLLGNFFCIGKRKRPEVTEPEEVASEEKLHKPPVDVGVDSRE  447
hMCT4   YMYVFILAGAEVLTSSLILLLGNFFCIRKKPKEPQPEV-AAAEEEKLHKPPADSGVDLRE  442

mMCT1   TMK--AAQSPQQH-SSGDPTEEESPV  493
hMCT1   VTK--AAESPDQKDTDGGPKEEESPV  500
mMCT4   VEHFLKAEPEKNGEVVHTPETSV---  470
hMCT4   VEHFLKAEPEKNGEVVHTPETSV---  465
```

Supplementary Fig. S1. Amino-acid sequence alignment of mouse and human MCT1s and MCT4s.

The secondary structure elements of mMCT1 are displayed, with functionally important amino acids interacting with mBSG (based on MD simulation) highlighted in green. Residues annotated by circles, a triangle, or diamonds participate in H-bond interactions connecting the mMCT1's NTD with mBSG's TM0 on the extracellular side, intramembrane, and on the intracellular side, respectively.

Supplementary Fig.S2

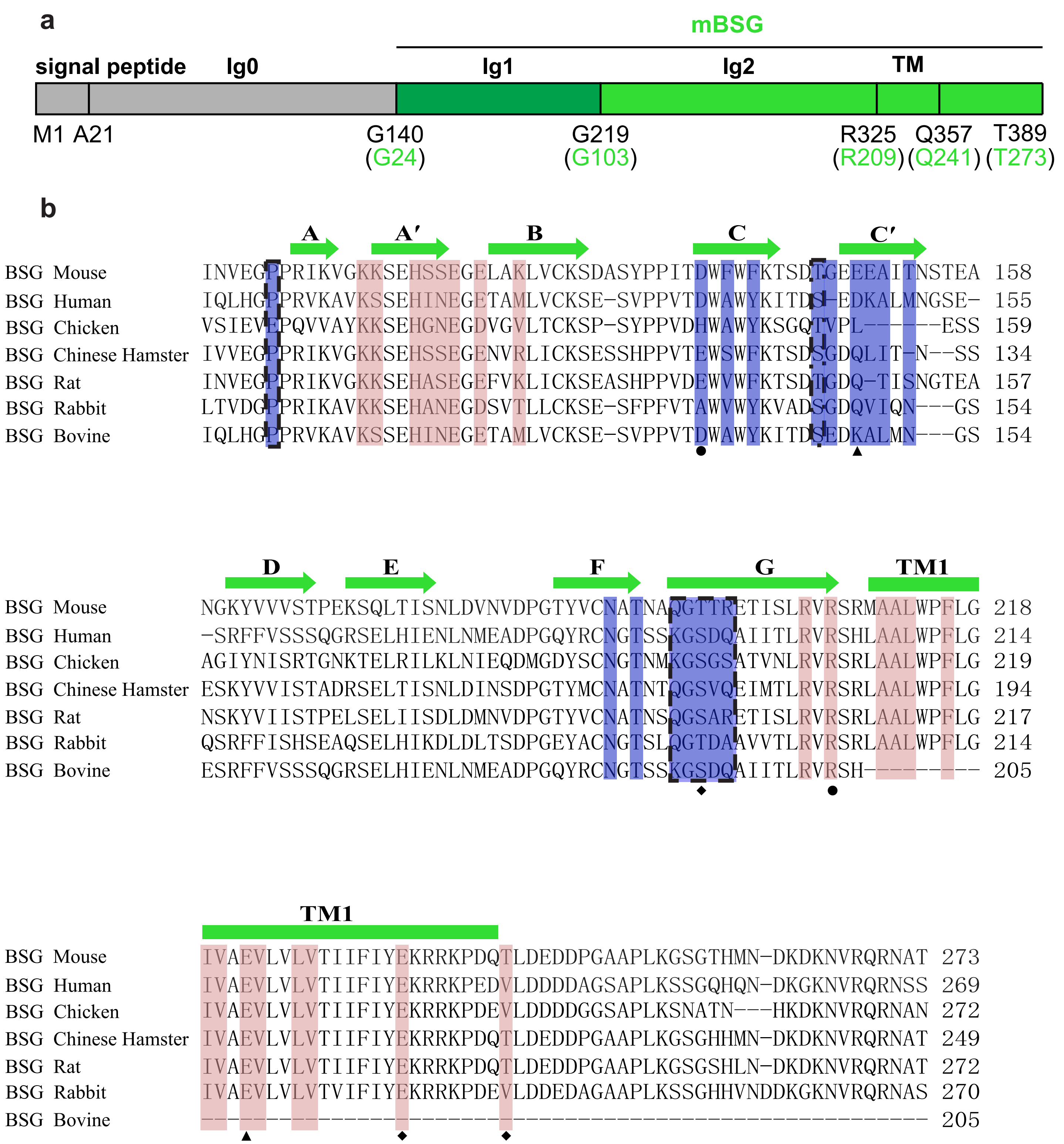

Supplementary Fig. S2. Annotations of the mouse basigin.

a Diagrammatic representation of mouse basigin with its key domains. Basigin has multiple isoforms due to differential splicing and transcription initiation sites[13]. Basigin-1 (BSG-1) is retina-specific, possessing three Ig domains, while the common form, Basigin-2 (BSG-2), has two Ig domains and is referred to simply as BSG.

b Sequence alignment of Ig2 domains and transmembrane domains from vertebrate basigins is shown. Secondary structure elements are indicated for mBSG. Amino acid residues colored in blue interact with 6E7F1Fab based on cryo-EM data. Unframed and dash-framed residues engage with the heavy and light chains of 6E7F1Fab, respectively. Residues D137, E149, and T197 are marked by a circle, triangle, and diamond, respectively, each contacting multiple residues of 6E7F1Fab. Residues colored in salmon are predicted by MD simulations to interact with mMCT1. Residues R207, E222, E234 and T242, marked by a circle, triangle, and diamonds, participate in H-bond interactions between mBSG's TM0 and mMCT1's NTD on the extracellular side, intramembrane, and on the intracellular side, respectively.

Supplementary Fig.S3

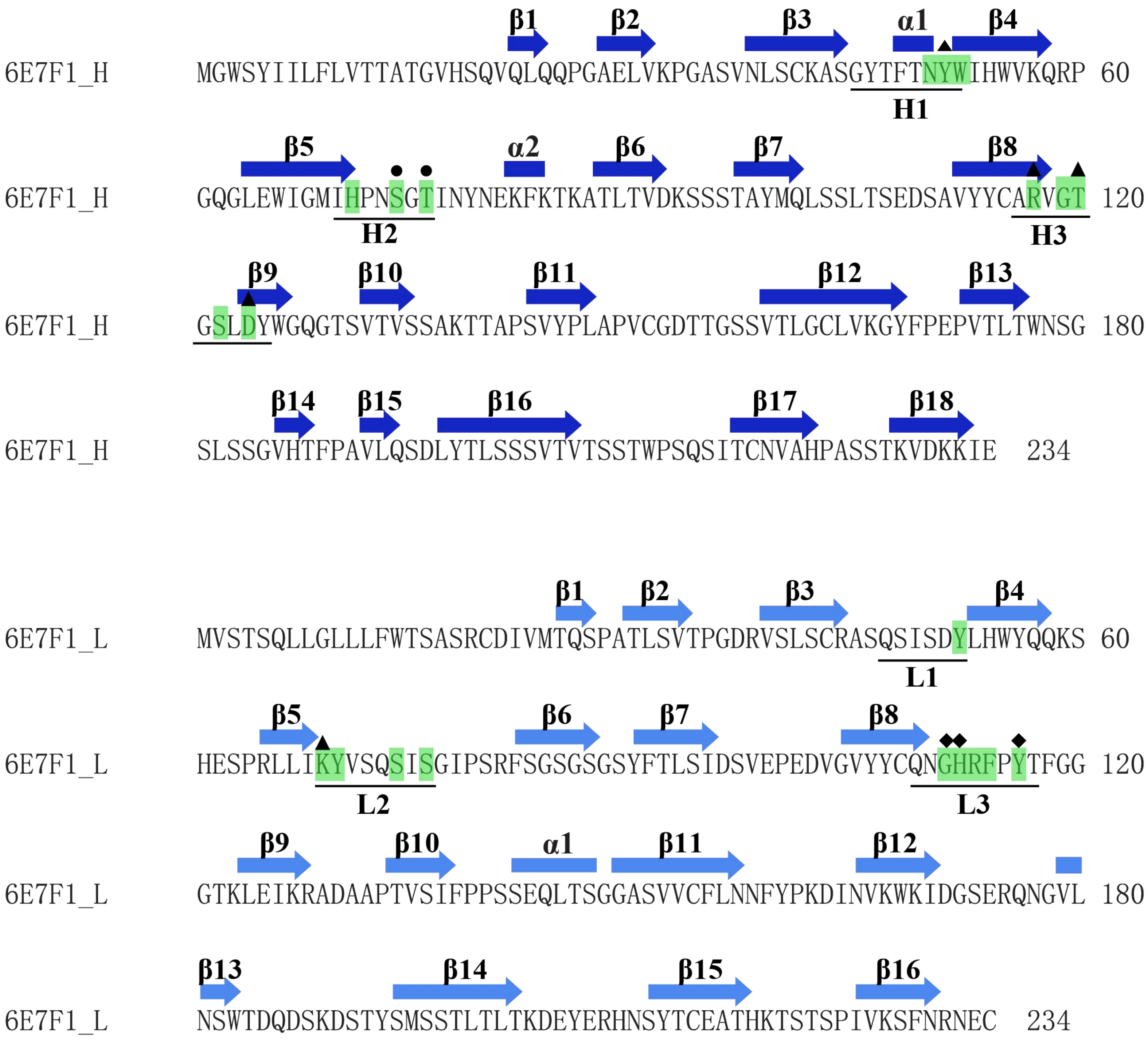

Supplementary Fig. S3. Annotations of the 6E7F1Fab.

Green-highlighted residues participate in interactions with mBSG. The CDRs 1-3 of the heavy and light chains are labeled as H1-H3 and L1-L3, respectively. Additionally, solid circles, triangles, and diamonds denote residues involved in polar interactions with mBSG residues D137, E149, and T197, respectively.

Supplementary Fig.S4

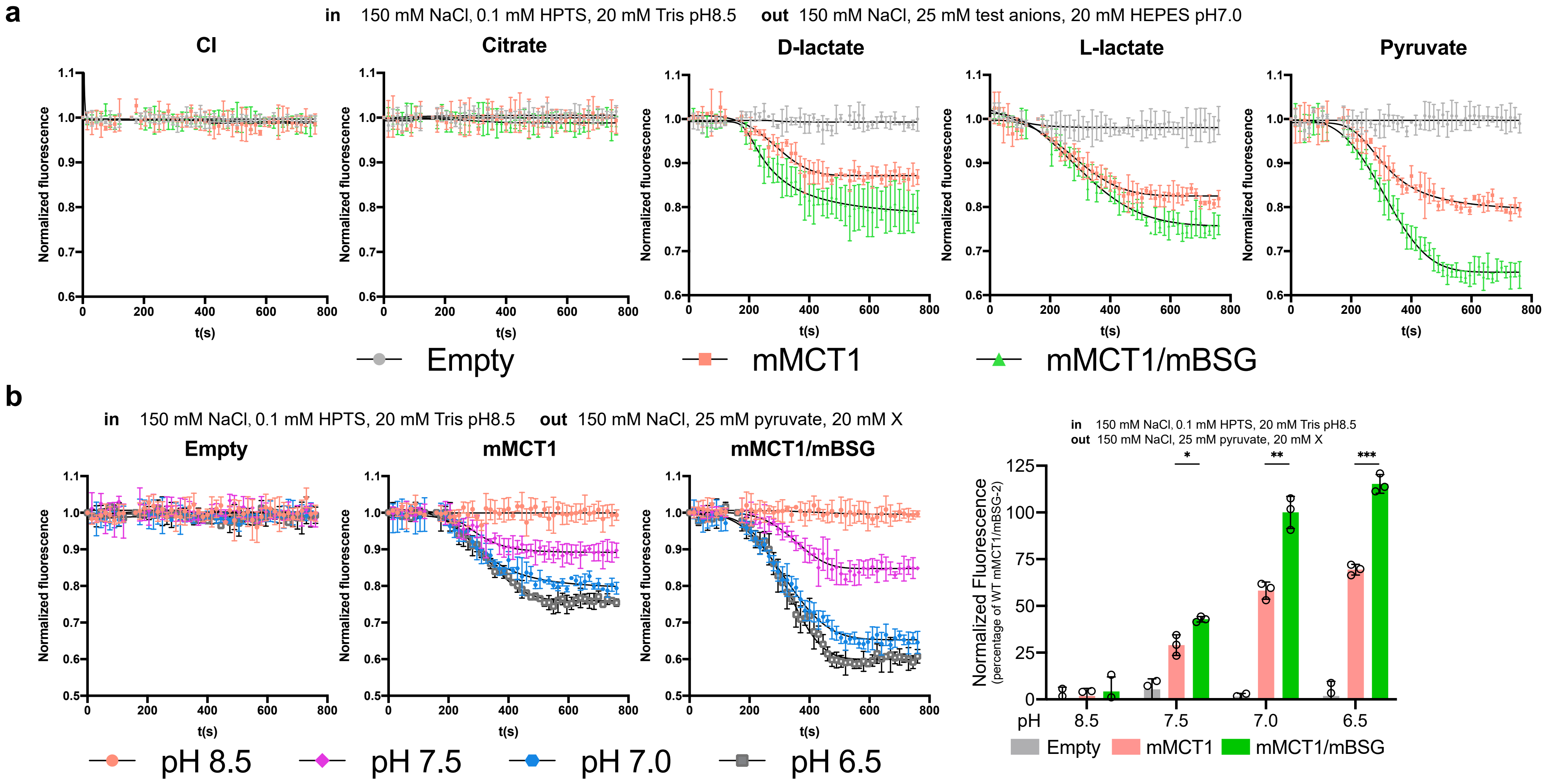

Supplementary Fig. S4. Transport activities of mMCT1 and mMCT1/mBSG characterized by fluorescence-based liposomal flux assays.

a Fluorescence traces for the test anions align with those in **Fig. 1a**. All test anions used are sodium salts.

b The mMCT1- or mMCT1/mBSG-mediated H^+/pyruvate influxes at various H^+ gradients. The left panel displays fluorescence traces, while the right panel shows quantifications. The intraliposomal pH remained constant at 8.5, and all normalized fluorescence traces are scaled to that of mMCT1/mBSG at pH 7.0.

Supplementary Fig.S5

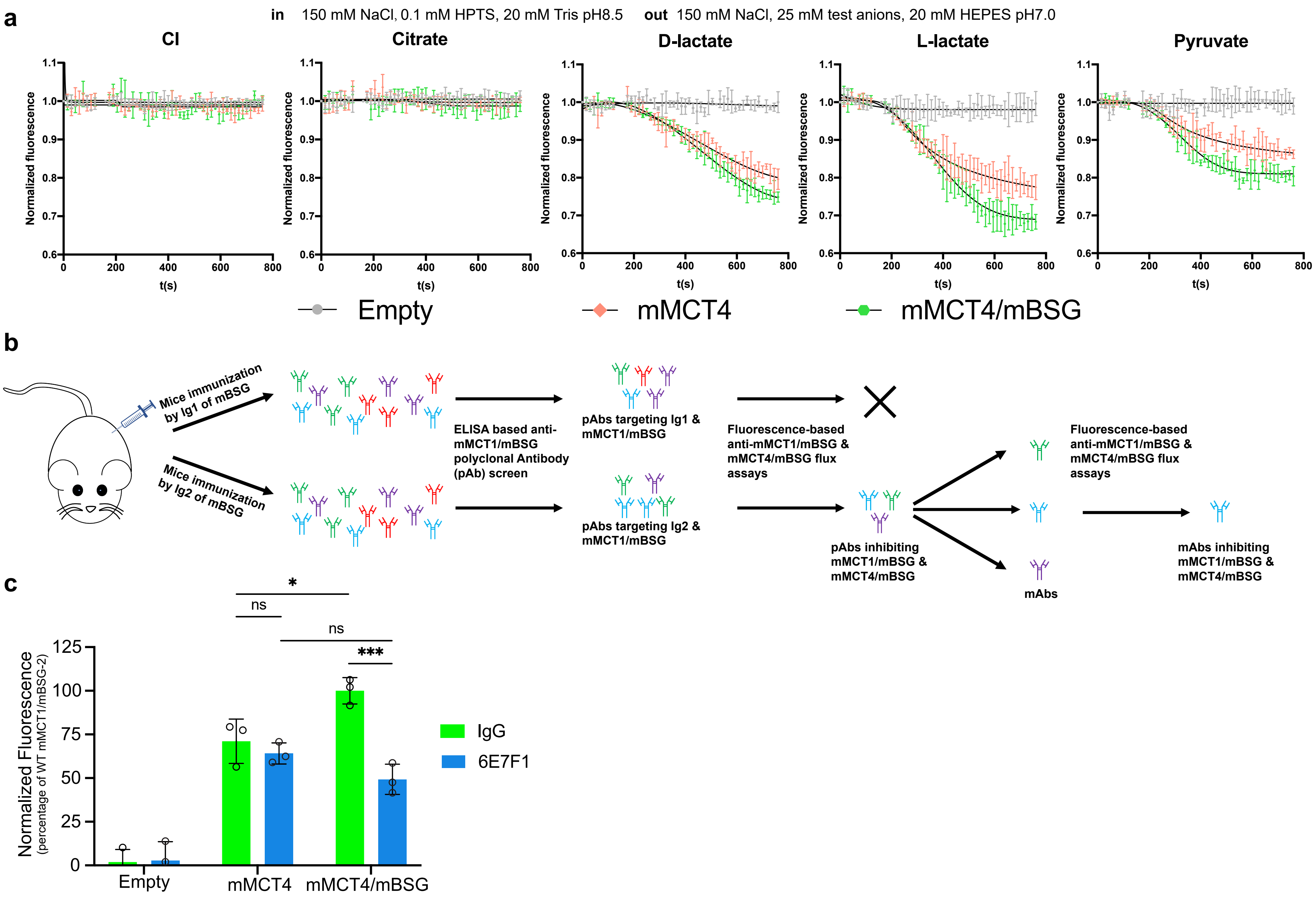

Supplementary Fig. S5. Transport activities of mMCT4 and mMCT4/mBSG characterized by fluorescence-based liposomal flux assays and the search protocol of anti-mBSG antibodies that inhibit mMCTs.

a Fluorescence traces for the test anions align with those in **Fig. 1b**. All test anions used are sodium salts.

b A schematic representation illustrating the screening process for antibodies that inhibit both mMCT1 and mMCT4. Initially, mice were inoculated with either the Ig1 or Ig2 domain of mBSG to generate polyclonal antibodies. These antibodies were then subjected to ELISA to select those that tightly bind to full-length mMCT1/mBSG. Subsequently, fluorescence-based liposomal flux assays were employed to identify antibodies inhibiting both mMCT1/mBSG and mMCT4/mBSG. Notably, only Ig2-specific antibodies demonstrated MCT inhibition. Finally, monoclonal antibodies were generated and re-screened using fluorescence-based liposomal flux assays to identify those effectively inhibiting MCTs.

c mMCT4- or mMCT4/mBSG-mediated H^+/L-lactate influx, in the presence of 0.6 μM IgG control or 6E7F1.

Supplementary Fig.S6

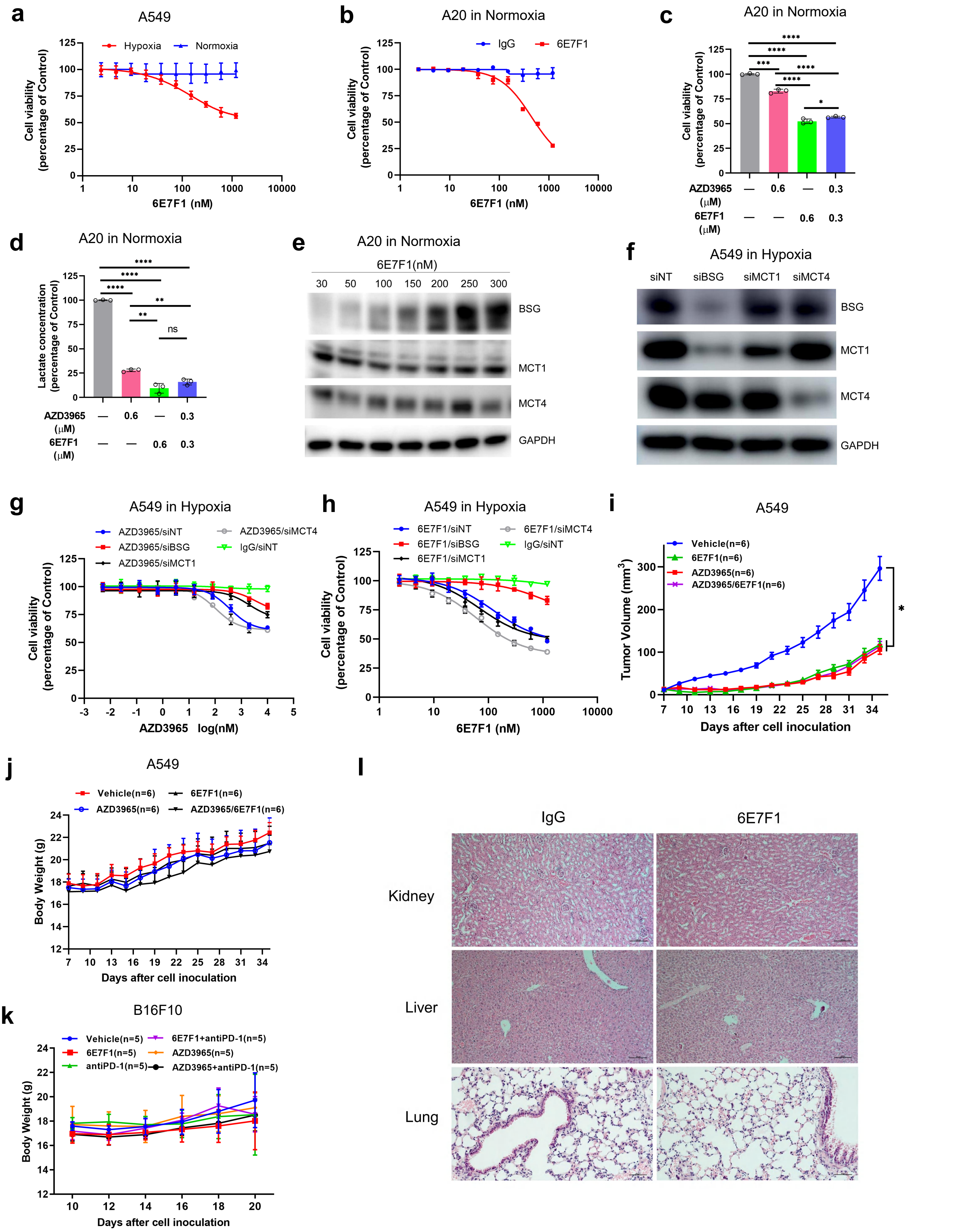

Supplementary Fig. S6. 6E7F1 suppresses the growth of human A549 and murine A20 tumor cells *in vitro* and *in vivo*.

a, b Determination of IC_{50} for the 6E7F1 antibody on A549 cells under both hypoxia and normoxia (**a**), and on A20 cells under normoxia (**b**).

c, d Measurement of cell viability (**c**) and extracellular lactate concentration (**d**) in A20 cell cultures treated with 6E7F1 and/or AZD3965 under normoxia.

e, f Western blot analysis of BSG, MCT1, and MCT4 protein levels in A20 cells (**e**) exposed to normoxia with increasing concentrations of 6E7F1, and in A549 cells (**f**) subjected to siRNA treatment under hypoxia.

g, h Viability measurements for siRNA-treated A549 cells exposed to either AZD3965 (**g**) or 6E7F1 (**h**) under hypoxia.

i, j The impact of 6E7F1 and AZD3965 on A549 xenograft tumor growth (**i**) and body weight (**j**) in immunocompromised BALB/c nude mice.

k The effect of 6E7F1, AZD3965, and/or PD-1 antibody on the body weight of immunocompetent C57BL/6 mice bearing B16F10 allograft tumors.

l H&E staining of the kidney, liver, and lung from mice treated with 6E7F1 antibody.

Supplementary Fig.S7

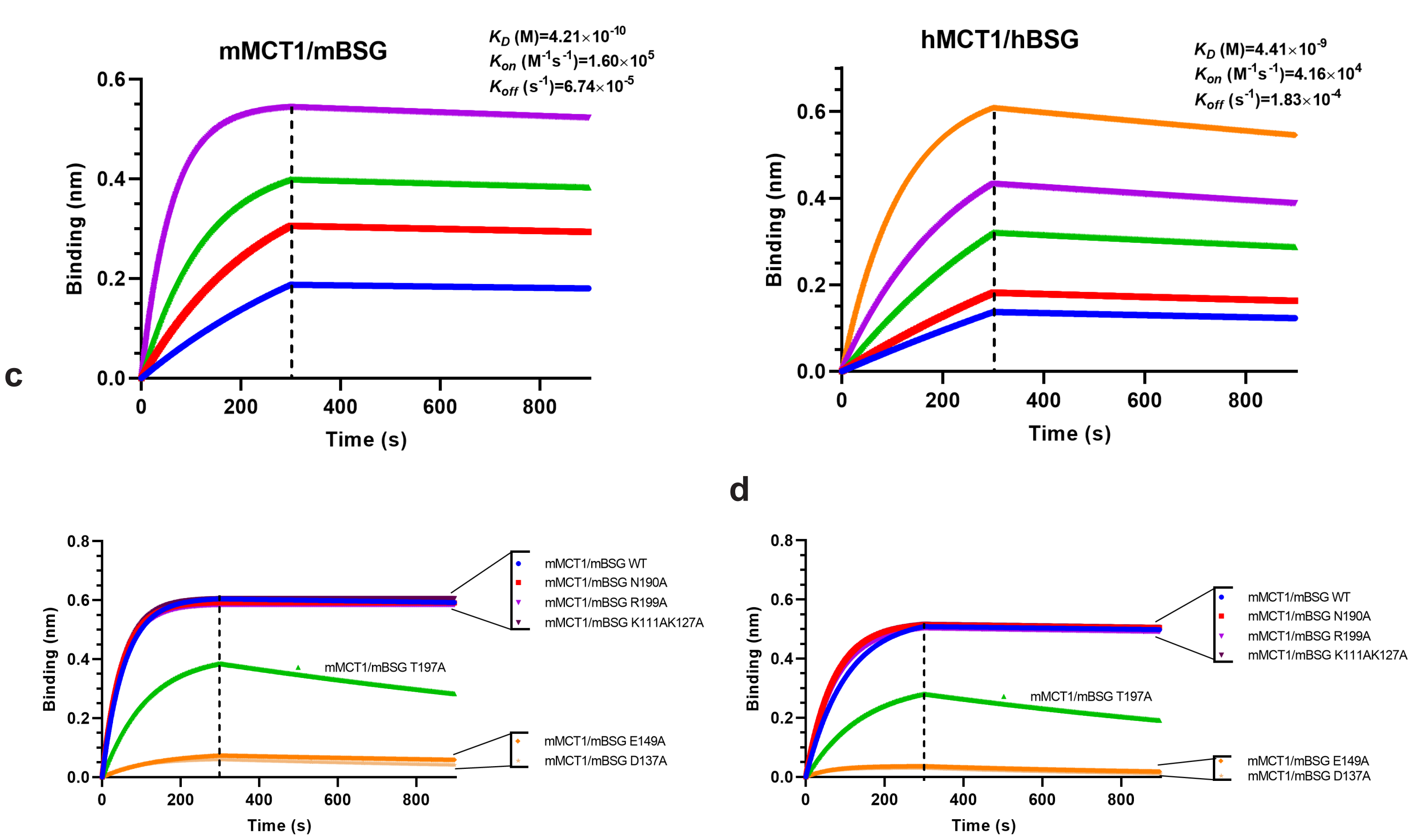

Supplementary Fig. S7. The binding affinities of 6E7F1 to WT mMCT1/mBSG, WT hMCT1/hBSG, and mMCT1/mBSG variants measured using Octet biolayer interferometry. The analysis utilized a 1:1 binding model, with purified 6E7F1 immobilized on Anti-Mouse IgG Fc Capture biosensors.

a, b Binding affinities of 6E7F1 to WT mMCT1/mBSG (**a**) and WT hMCT1/hBSG (**b**). The analytes WT mMCT1/mBSG and hMCT1/hBSG were serially diluted from 100 to 12.5 nM (**a**) and from 200 to 12.5 nM (**b**).

c, d The binding affinities of 6E7F1 to WT mMCT1/mBSG and its variants. The analytes, including WT mMCT1/mBSG and each variant, were tested at concentrations of 100 nM (**c**) and 50 nM (**d**).

Supplementary Fig.S8

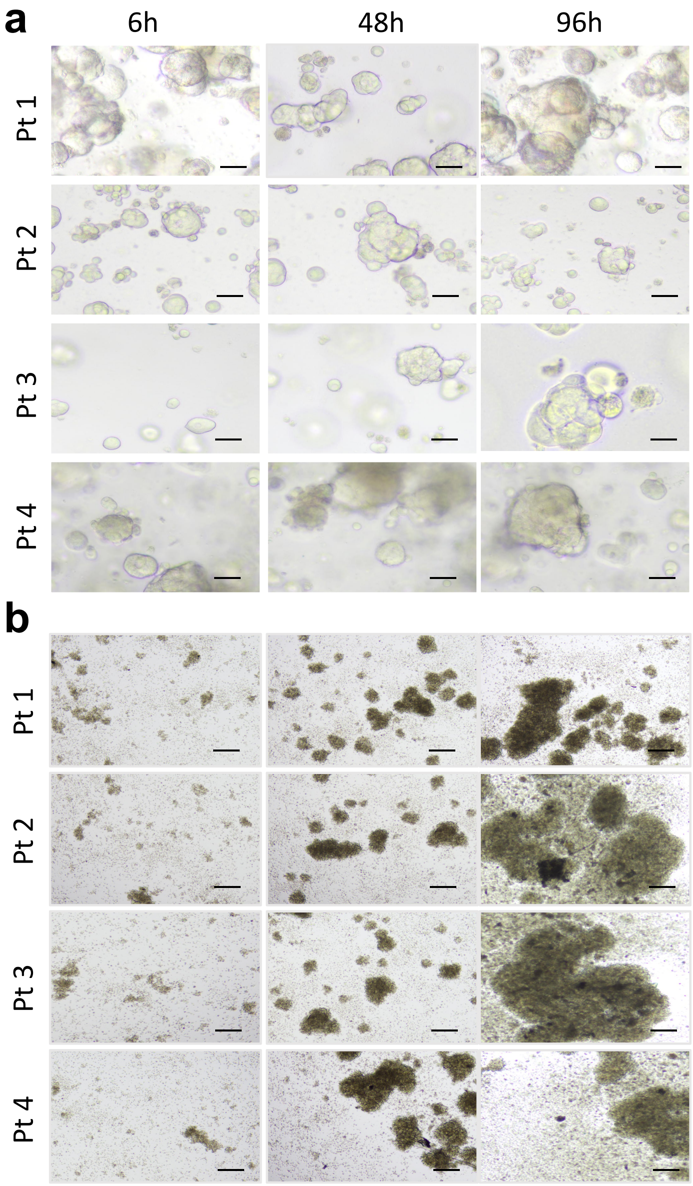

Supplementary Fig. S8. The growth of organoids (a) and immune cells (b) under hypoxia. Representative images illustrating the growth dynamics of organoids and immune cells over time, captured at intervals from 6 hours (6h) to 96 hours (96h). Scale bar = 100 µm.

Supplementary Fig.S9

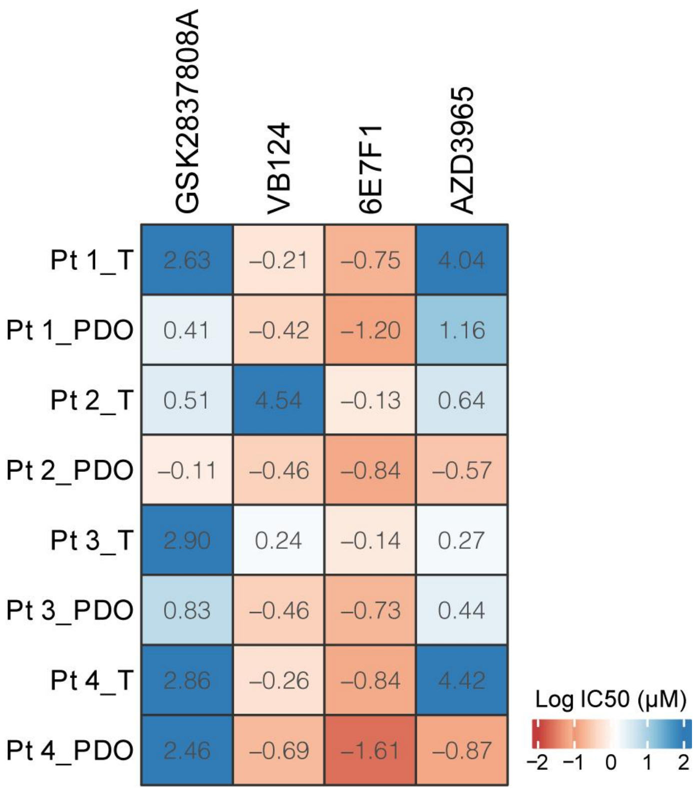

Supplementary Fig. S9. The heatmap depicting the drug sensitivity of NSCLC-PDOs and their corresponding T cells to various drugs. The analysis aims to determine the IC50 concentration for the organoids while ensuring T cell viability. The color scale represents the average Log (IC50) values.

Supplementary Fig.S10

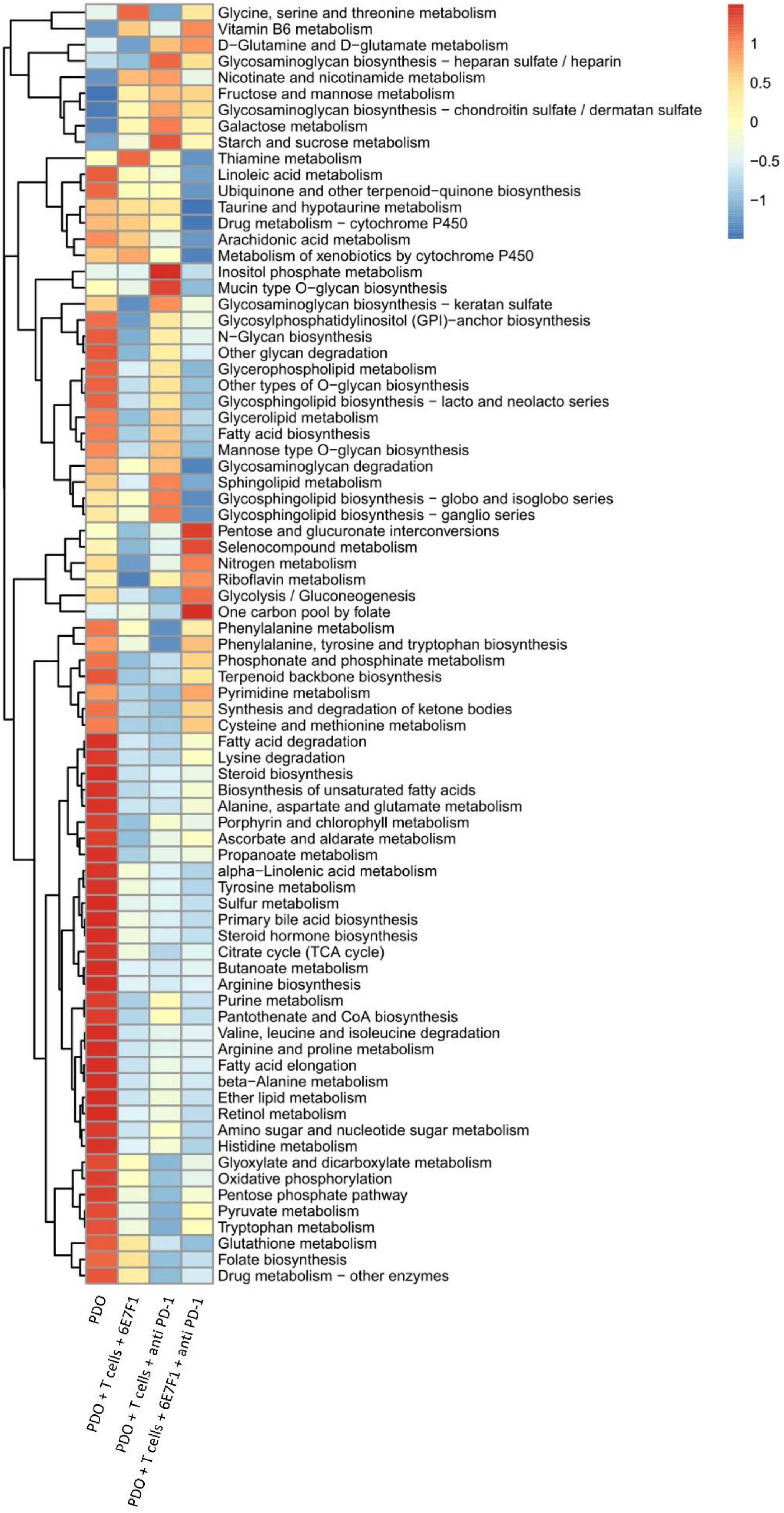

Supplementary Fig. S10. The heatmap displaying metabolic pathway scores for tumor epithelial cells under specified treatments. Rows: metabolic pathways; columns: treatment groups. Color key indicates metabolite expression value: dark blue: lowest; dark red: highest.

Supplementary Fig.S11

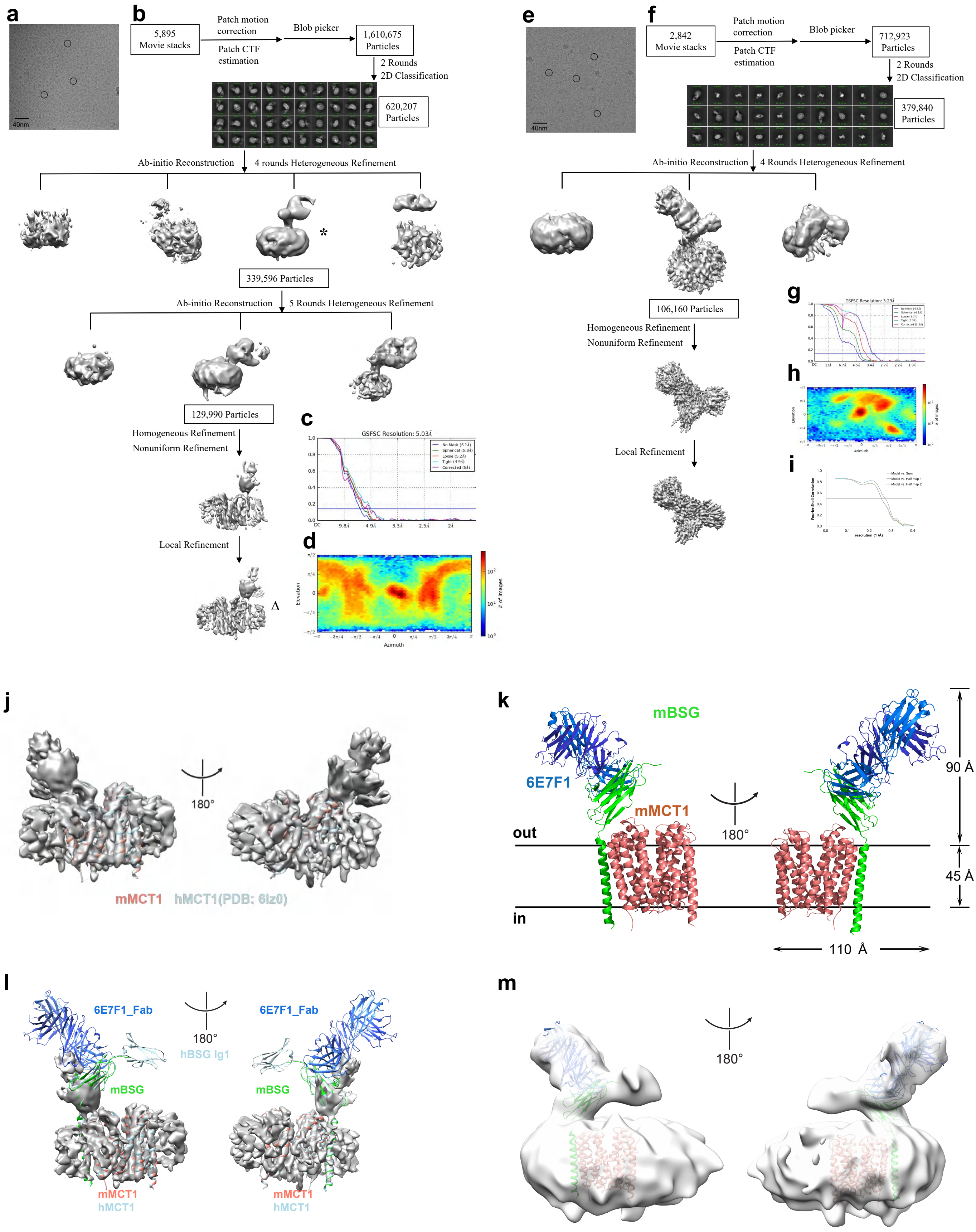

Supplementary Fig. S11. Cryo-EM structure determination of the mMCT1/mBSG/6E7F1Fab complex at resolutions of 5.0 Å and 3.2 Å, respectively.

a, e Representative cryo-EM micrographs of the mMCT1/mBSG/6E7F1Fab complex in the 5.0 Å (**a**) and 3.2 Å (**e**) data sets. Circles indicate individual particles.

b, f Cryo-EM data processing flowcharts for the mMCT1/mBSG/6E7F1Fab structures at 5.0 Å (**b**) and 3.2 Å (**f**), including particle picking, classification, and 3D refinement.

c, g Fourier shell correlations (FSC) of the final 3D reconstruction following gold standard refinement. FSC curves are plotted both before and after masking.

d, h Angular distribution heatmaps of particles used for the refinement.

i Cross-validation FSC curves for the refined model versus the half maps (half maps 1 and 2) and the summed map.

j 3D EM reconstruction of the transmembrane portions of the mMCT1/mBSG/6E7F1Fab complex at 5.0 Å. The transmembrane regions (TMs) of mMCT1 are superimposed on the corresponding TMs of hMCT1 (PDB code: 6LZ0) to indicate their structural similarity.

k Overall structure of the mMCT1/mBSG complex bound by 6E7F1Fab in the outward

conformation. The protein complex is constructed by two segments: a 5.0 Å transmembrane segment containing 12 transmembrane regions (TMs) from mMCT1 and TM0 of mBSG, and a 3.2 Å extracellular segment that includes the Ig2 domain of mBSG and 6E7F1Fab. The membrane boundary is also indicated.

l, m The assembled mMCT1/mBSG-2/6E7F1Fab complex docked into two densities in panel B: Δ (5.0-Å resolution, grey, L) and * (~10.0-Å resolution, grey, M).

Supplementary Fig.S12

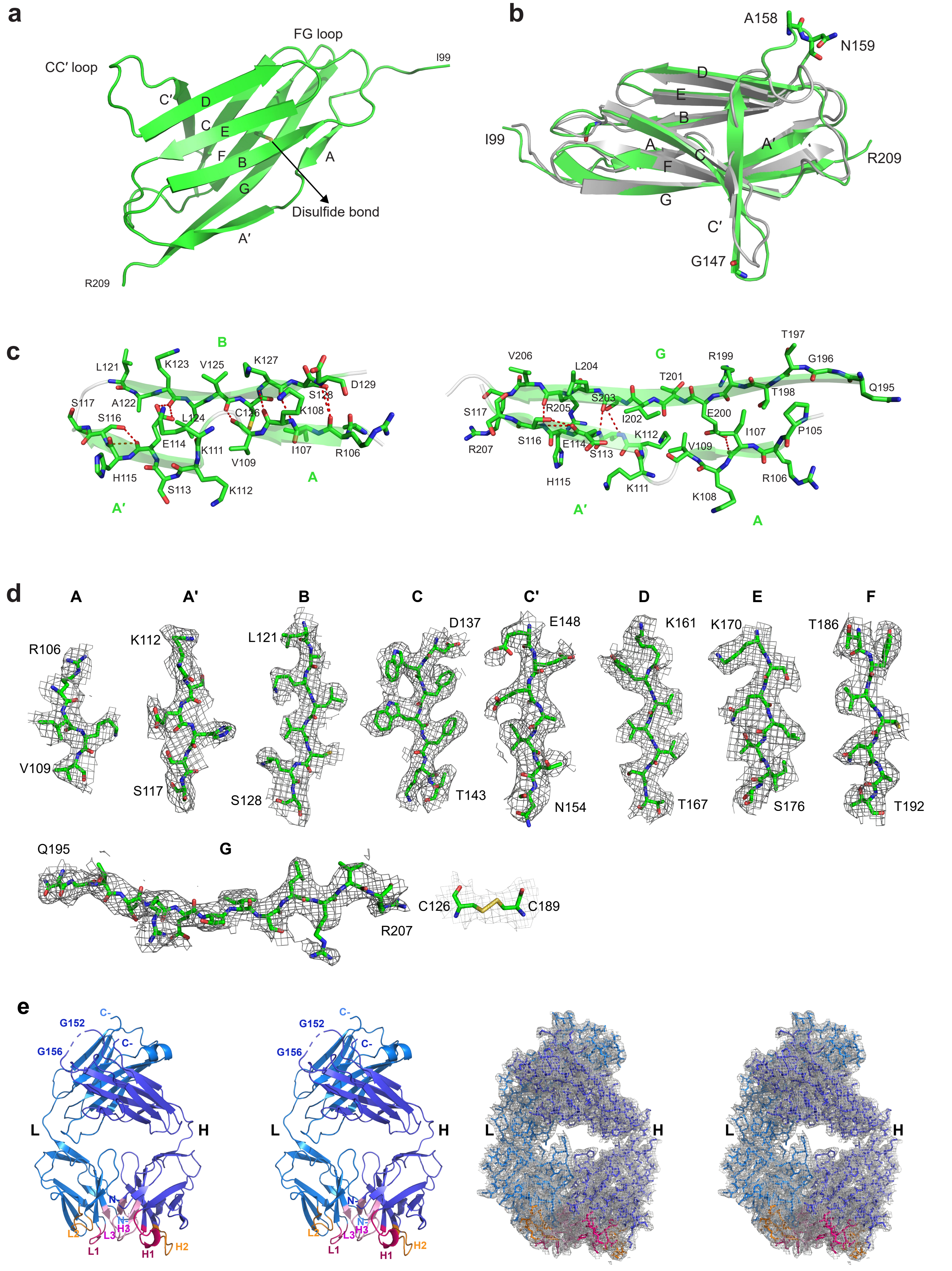

Supplementary Fig. S12. Atomic models of mBSG and 6E7F1Fab.

a Overall structure of the Ig2 domain of mBSG, highlighting all eight β strands, the disulfide bond between strands B and F, and the CC′ and FG loops involved in interactions with 6E7F1Fab.

b Superimposition of the Ig2 domains of mBSG (green) and hBSG (grey, PDB code: 3B5H) shows structural similarity with an r.m.s.d. of 1.6 Å for 104 aligned Cα atoms. Extra residues G147, A158, and N159 of mBSG are shown as sticks.

c The I99-G119 segment, containing strands A and A′, interacts with strand B (left) via H-bonding and strand G (right) via H-bonding and hydrophobic interactions.

d The 3.2 Å cryo-EM density (grey, 5.0 σ) superimposed on the refined Ig2 domain structure of mBSG, with labeled secondary structure elements and disulfide bonds.

e Stereoview of the 6E7F1Fab atomic model in ribbon representation (left) and the stereoview of the 3.2 Å cryo-EM density (right, at 5.0 σ). A small electron density break is observed between G152 and G156 in the heavy chain. CDRs 1-3 of the heavy and light chains are labeled as H1-H3 and L1-L3, respectively.

Supplementary Fig.S13

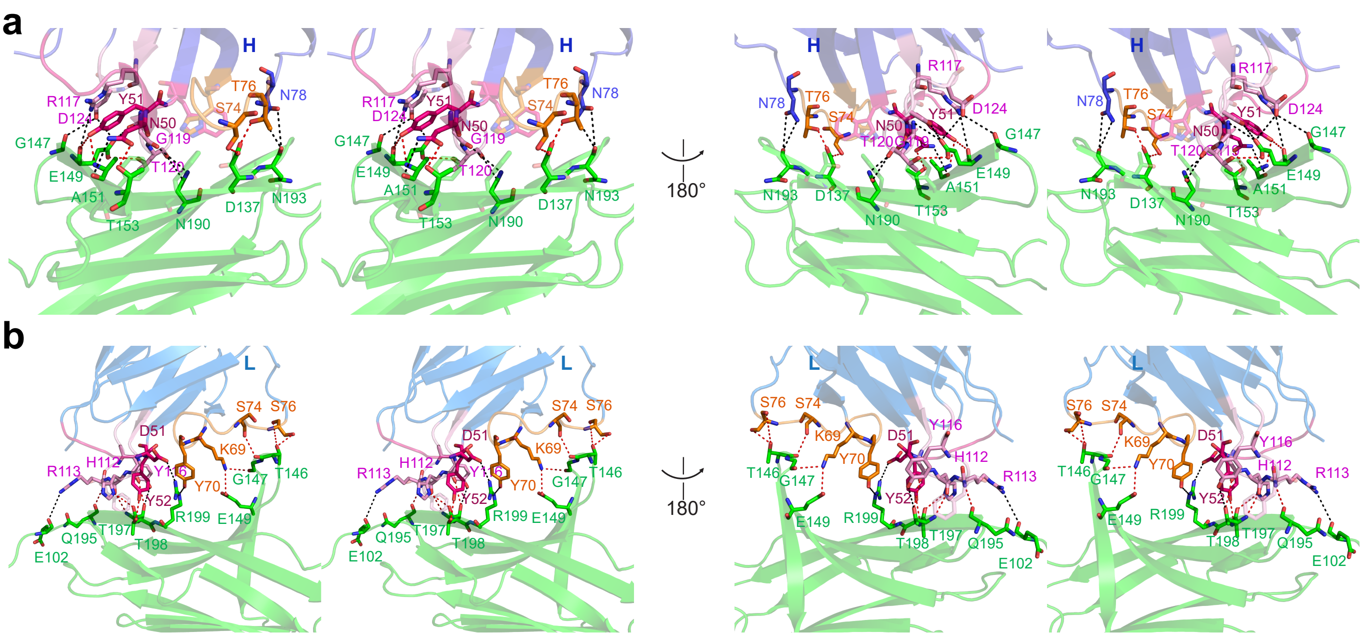

Supplementary Fig. S13. Stereoviews showing the interactions between 6E7F1Fab's heavy (a) and light (b) chains with the Ig2 domain of mBSG. The heavy and light chains of 6E7F1Fab are depicted in dark blue and light blue, respectively, while mBSG is shown in green.

Supplementary Fig.S14

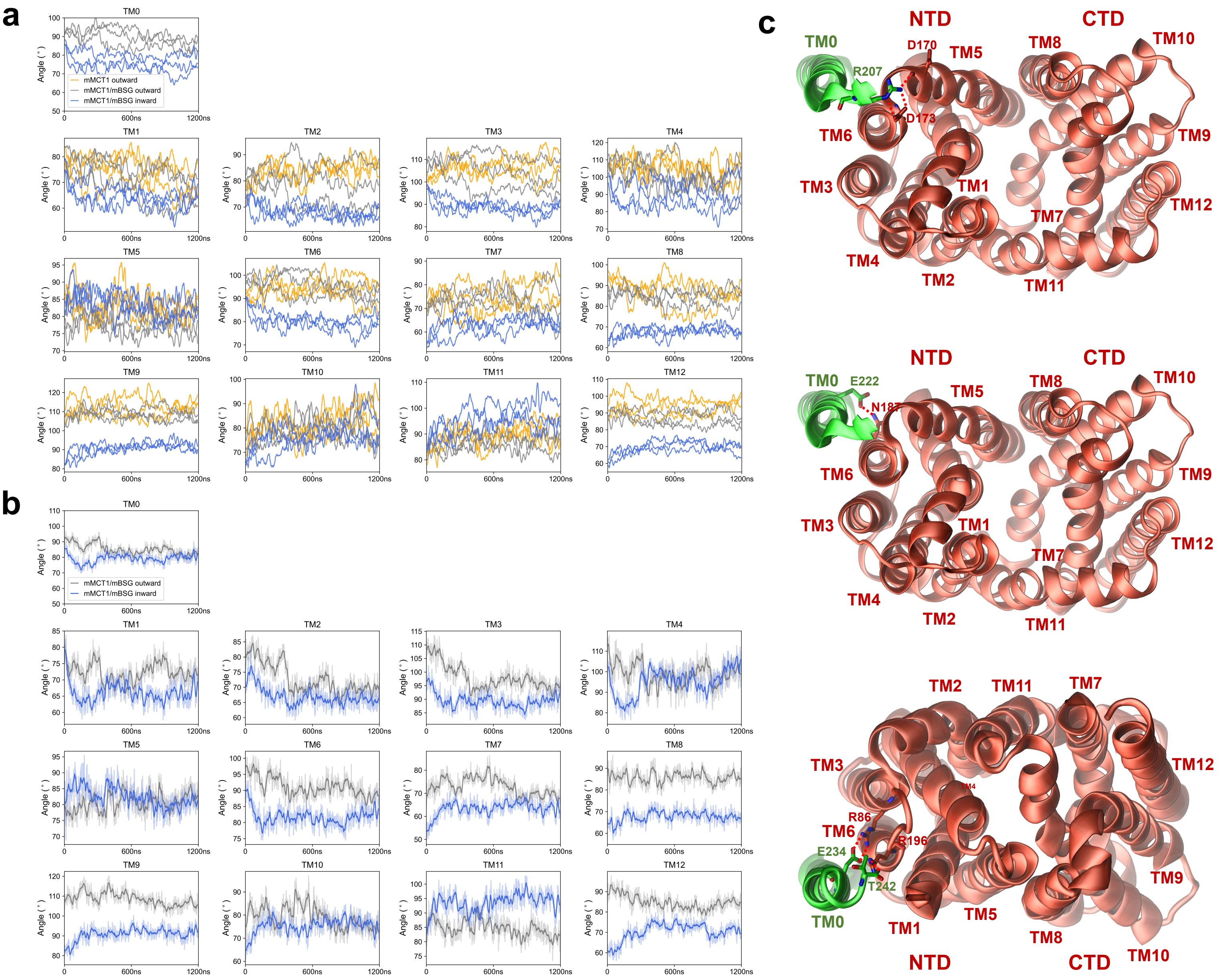

Supplementary Fig. S14. Time-evolved conformational changes of TMs over MD trajectories for outward-open mMCT1, as well as outward- and inward-open mMCT1/mBSGs.

a Time-evolved changes of TM angles relative to the membrane surface over a set of three independent trajectories for each structure.

b A representative trajectory in **(a)** showing changes in TM angles relative to the membrane surface during the outward- to inward-facing transition of the mMCT1's NTD in a simulation initiated with the outward-facing mMCT1/mBSG. These trajectories are the same as those in **Fig. 6e**, **f**.

c A representative snapshot of mMCT1/mBSG showing three sets of H-bonds linking mBSG's TM0 to the mMCT1's NTD. The top and middle panels display H-bonds at the extracellular side and within the membrane, respectively, while the bottom panel shows H-bonds on the intracellular side.

Supplementary Fig.S15

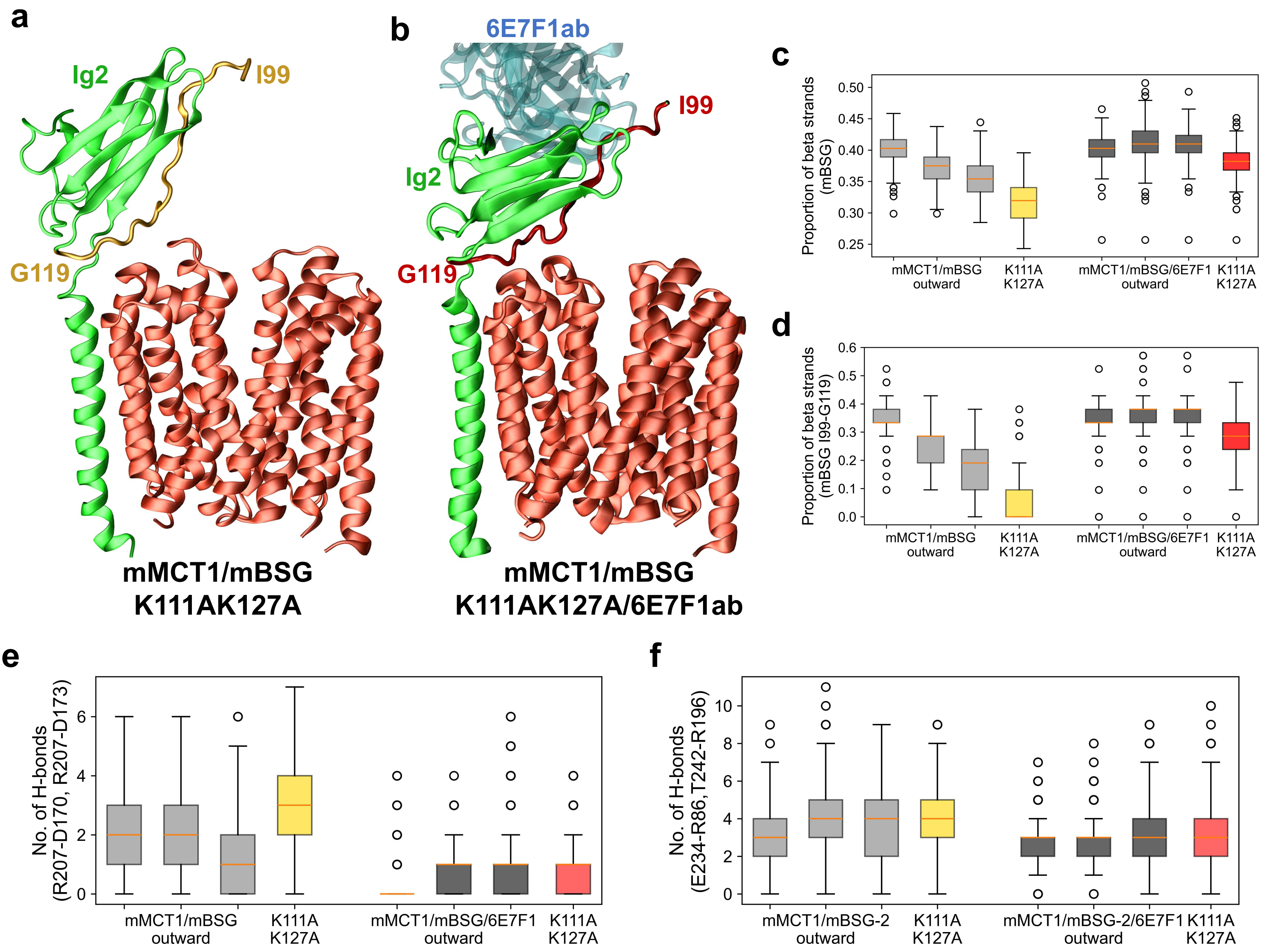

Supplementary Fig. S15. The mMCT1/mBSG (K111A/K127A) variant shows greater conformational flexibility in the Ig2 domain and increased hydrogen-bond interactions between mBSG's TM0 and mMCT1's NTD compared to the WT mBSG. The boxplots in (**c-f**) show the distribution of data from the first quartile (Q1) to the third quartile (Q3), with an orange line at the median. The whiskers extend from the box by 1.5 times the interquartile range (IQR). Outliers beyond the whiskers are represented as individual points.

a, b Representative snapshots from trajectories of the mMCT1/mBSG (K111A/K127A) variant (**a**) and mMCT1/mBSG (K111A/K127A)/6E7F1Fab (**b**). The mBSG (K111A/K127A) variant exhibits a loop conformation for the I99-G119 segment, while in the WT mBSG, this segment forms strands A and A′ in simulation trajectories.

c, d Boxplots depicting the β-strand proportions within the Ig2 domain (**c**) and its I99-G119 segment (**d**) across simulation trajectories. The mBSG (K111A/K127A) variant consistently exhibits lower β-strand proportions in both the Ig2 domain and I99-G119 segment compared to the WT mBSG, no matter its binds to 6E7F1Fab (red) or not (yellow).

e, f Boxplots showing the number of extracellular (e) and intracellular (f) H-bonds between mBSG's TM0 and mMCT1's NTD over MD trajectories. In the absence of 6E7F1, the mMCT1/mBSG (K111A/K127A) variant (yellow) exhibits more extracellular H-bonds than the WT mMCT1/mBSG, while their intracellular H-bonds remain similar. Upon 6E7F1 binding, both extracellular and intracellular H-bonds drop to similar levels for the mMCT1/mBSG (K111A/K127A) variant (red) and the WT mMCT1/mBSG.

Supplementary Fig.S16

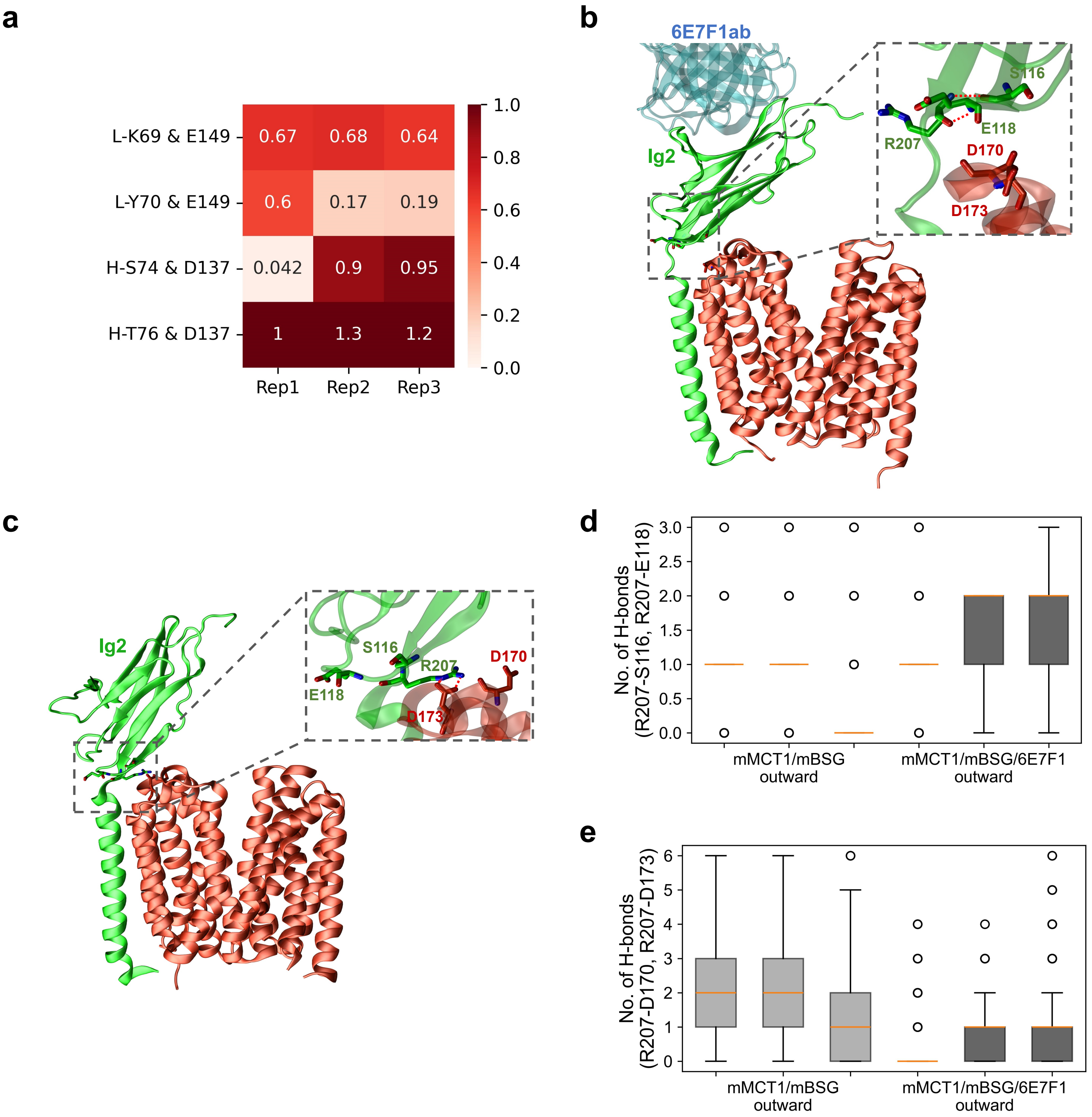

Supplementary Fig. S16. The binding of 6E7F1Fab to mBSG enhances H-bond interactions between the anchor and strand A′ in the Ig2 domain of mBSG, but impairs H-bond interactions between mBSG's TM0 and mMCT1's NTD.

a Heatmap showing the frequency of hydrogen bonds between BSG and the antibody during dynamic simulations of the outward conformation. Each row represents a pair of hydrogen bonds, and each column represents a parallel simulation trajectory. The color of the heatmap indicates the average number of hydrogen bonds between two residues over a 1200 ns trajectory.

b, c Representative snapshots from trajectories showing H-bond interactions between the anchor's R207 and strand A′ (S116 and E118) within mBSG (**b**), and between the anchor's R207 of mBSG and the NTD (D170 and D173) of mMCT1 (**c**).

d, e Boxplots showing H-bond counts between the anchor' R207 and strand A′ (S116 and E118) within mBSG (**d**), and between the mBSG's R207 and mMCT1's NTD (D170 and D173) (**e**).

Supplementary Fig.S17

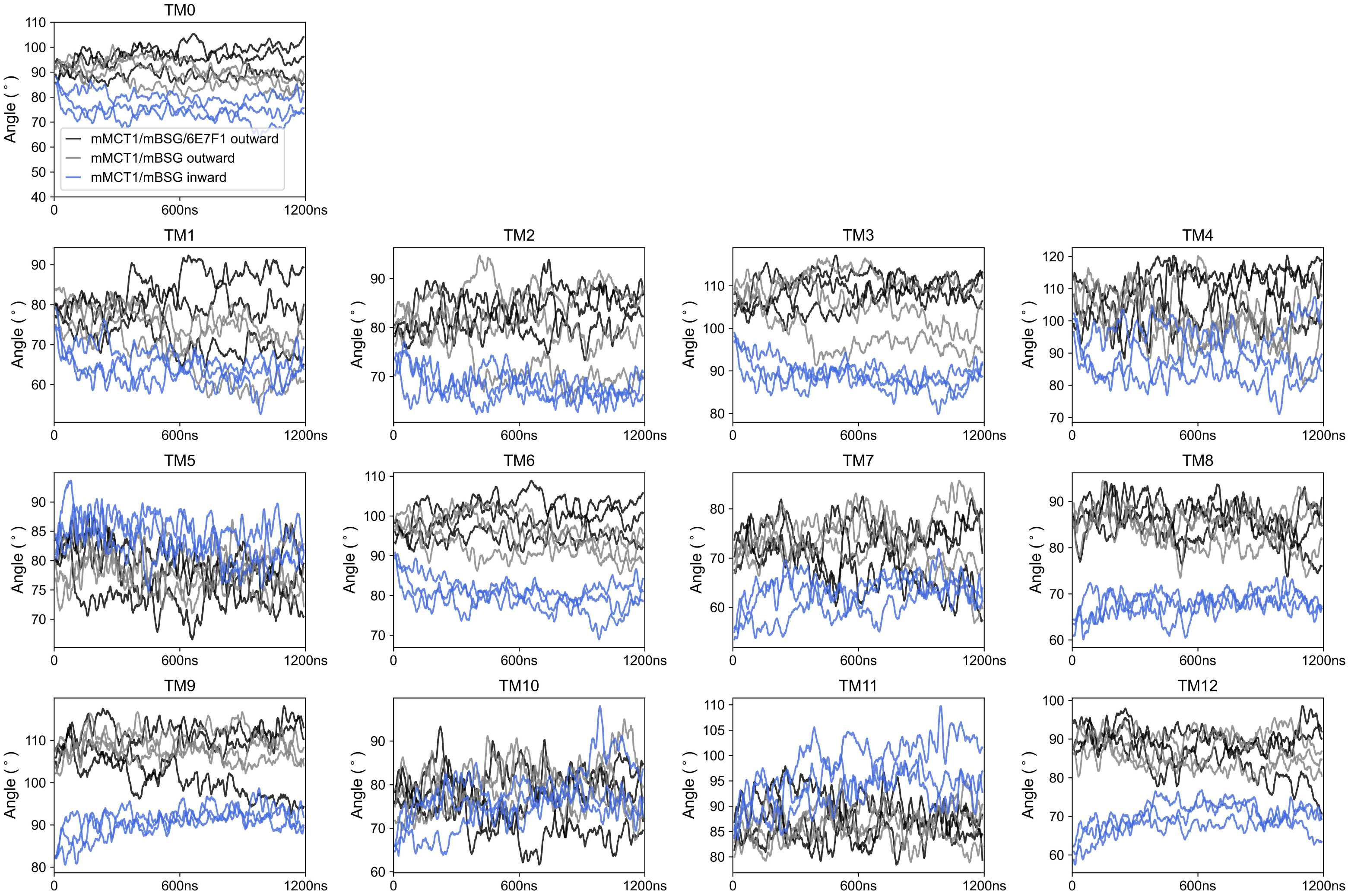

Supplementary Fig. S17. The time-evolved conformational changes for TMs. Changes in the angles of all TMs in the NTD of mMCT1 relative to the membrane surface during 1200 ns simulation trajectories for the outward-facing mMCT1/mBSG/6E7F1Fab, as well as for both the outward- and inward-facing mMCT1/mBSG.

Supplementary Table S1

	5.0 Å map	3.2 Å map
**Data collection and processing**		
Microscope	FEI Titan krios	FEI Titan krios
Detector	Gatan K2 Summit + GIF	Gatan K2 Summit + GIF
Magnification	105,000	105,000
Voltage (kV)	300	300
Electron exposure ($e^-/Å^2$)	75	75
Defocus range (μm)	-1.0 ~ -2.0	-1.0 ~ -2.0
Pixel size (Å)	0.82	0.84
Symmetry imposed	C1	C1
Number of movies	5,895	2,842
Initial particle images (no.)	1,610,675	712,923
Final particle images (no.)	129,990	106,160
Map resolution (Å)	5.0	3.2
Sharpening B-factor ($Å^2$)	-125.0	-77.3
FSC threshold	0.143	0.143
**Model composition**		
Non-hydrogen atoms		4137
Protein residues		541
Ligands		0
**B-factor and R.m.s. deviations**		
Mean B-factor protein atoms ($Å^2$)		39.46
Rmsd bonds (Å)		0.006
Rmsd bond angles (°)		1.045
**Validation**		
MolProbity score		1.81
Clash score		5.04
Rotamer outliers (%)		0
Cβ outliers (%)		0
CaBLAM outliers (%)		3.43
**EMRinger score**		
Favored (%)		90.24
Allowed (%)		9.76
Outliers (%)		0

Supplementary Movie 1. Structural changes in mMCT1/mBSG over a 1200 ns trajectory.

Supplementary Movie 2. Structural changes in mMCT1/mBSG/6E7F1Fab over a 1200 ns trajectory.
